# Microfluidic technologies for lipid vesicle generation

**DOI:** 10.1039/d4lc00380b

**Published:** 2024-09-04

**Authors:** Yu Cheng, Callum D. Hay, Suchaya M. Mahuttanatan, James W. Hindley, Oscar Ces, Yuval Elani

**Affiliations:** a Institute of Chemical Biology, Molecular Sciences Research Hub, Imperial College London London UK j.hindley14@imperial.ac.uk o.ces@imperial.ac.uk y.elani@imperial.ac.uk; b Department of Chemistry, Molecular Sciences Research Hub, Imperial College London London UK; c Department of Chemical Engineering, Imperial College London London UK

## Abstract

Encapsulating biological and non-biological materials in lipid vesicles presents significant potential in both industrial and academic settings. When smaller than 100 nm, lipid vesicles and lipid nanoparticles are ideal vehicles for drug delivery, facilitating the delivery of payloads, improving pharmacokinetics, and reducing the off-target effects of therapeutics. When larger than 1 μm, vesicles are useful as model membranes for biophysical studies, as synthetic cell chassis, as bio-inspired supramolecular devices, and as the basis of protocells to explore the origin of life. As applications of lipid vesicles gain prominence in the fields of nanomedicine, biotechnology, and synthetic biology, there is a demand for advanced technologies for their controlled construction, with microfluidic methods at the forefront of these developments. Compared to conventional bulk methods, emerging microfluidic methods offer advantages such as precise size control, increased production throughput, high encapsulation efficiency, user-defined membrane properties (*i.e.*, lipid composition, vesicular architecture, compartmentalisation, membrane asymmetry, *etc.*), and potential integration with lab-on-chip manipulation and analysis modules. We provide a review of microfluidic lipid vesicle generation technologies, focusing on recent advances and state-of-the-art techniques. Principal technologies are described, and key research milestones are highlighted. The advantages and limitations of each approach are evaluated, and challenges and opportunities for microfluidic engineering of lipid vesicles to underpin a new generation of therapeutics, vaccines, sensors, and bio-inspired technologies are presented.

## Abstract 2

Encapsulating molecular materials in lipid vesicles *via* microfluidics provides control over size, morphology, and compartmentalisation, enabling their use in cell biology research and for applications in biomedicine, synthetic biology, and beyond.

## Graphical abstract

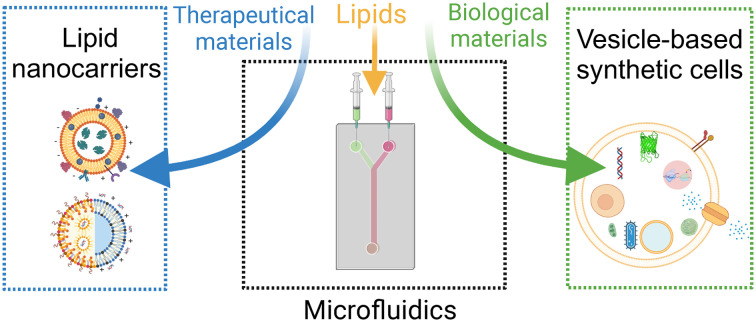

## 1 Introduction

### 1.1 Lipids and lipid vesicles

**Fig. 1:**
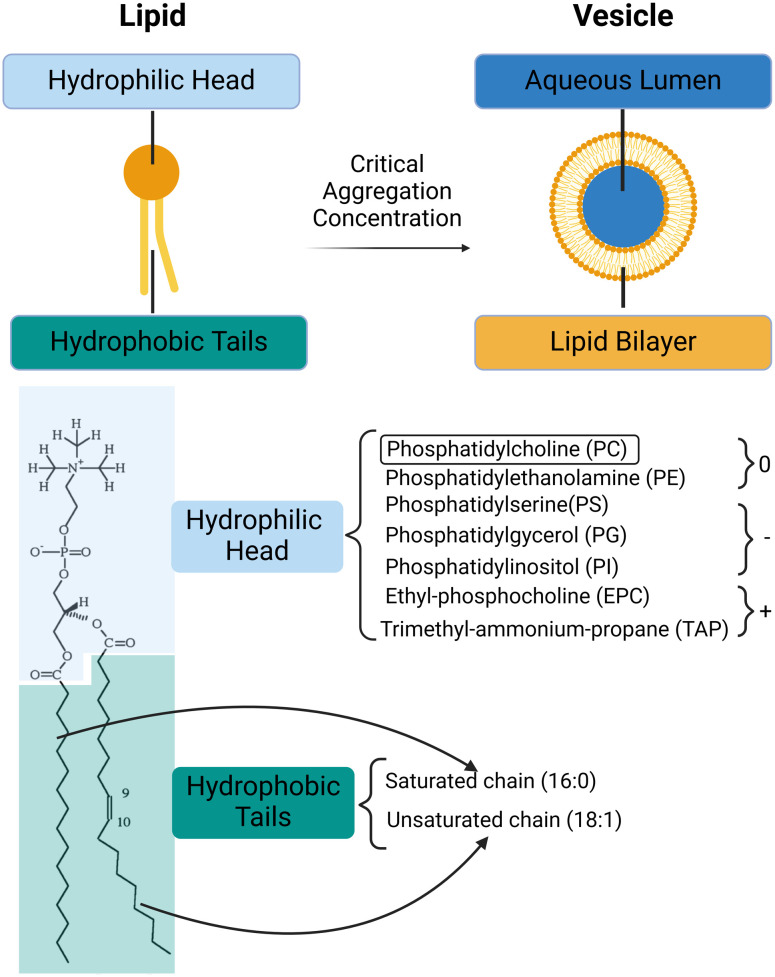
Lipids and vesicles. Top panel: Lipid self-assembly into vesicles is driven by the hydrophobic effect, minimising the interactions between hydrophobic tails and aqueous solution. Bottom panel: Typical lipid structure (POPC) is shown on the left. Common hydrophilic head groups of lipids and their charges at a physiological pH are listed. The hydrophobic tails of lipids can be saturated or unsaturated. For instance, the POPC lipid has one saturated 16 : 0 chain (16 carbons and 0 double bonds) and one unsaturated 18 : 1 chain (18 carbons and 1 double bond). 9 and 10 on the unsaturated tail of POPC are the carbons, between which the double bond locates.

Vesicles (or liposomes) are membrane-bound capsules which have an aqueous volume compartmentalised by one or more lipid bilayers.^1^ They can be viewed as self-enclosed three-dimensional supramolecular assemblies which are formed by the self-assembly of lipids^2^ (Fig. 1). Lipids are amphiphilic molecules composed of hydrophobic tails and hydrophilic heads; when mixed with water, the hydrophobic effect drives their self-assembly into lipid bilayers, which close up to form lipid vesicles.^3^ In an aqueous environment containing lipids above the critical aggregation concentration, hydrophobic tails of lipids rearrange so that they are screened by the hydrophilic head groups, preventing their unfavourable interaction with water and maximizing the entropy of water. The hydrophilic heads contact the exterior and interior aqueous environments, and the resulting spherical bilayer membrane compartmentalises an aqueous core. The shape of the lipid, influenced by the relative sizes of its hydrophilic head and hydrophobic tail, determines the phase of lipid assembly. Lipids with head and tail volumes that are approximately equal typically favour the formation of bilayers. However, lipids with different shapes may organise into various other self-assembled structures (Table 1).^4^

**Table 1:** Abbreviations of lipids

DDAB	Dimethyldioctadecyl-ammoniumbromide
DGS-NTA(Ni)	1,2-Dioleoyl-*sn-glycero*-3-[(*N*-(5-amino-1-carboxypentyl) iminodiacetic acid) succinyl] (nickel salt)
DMG-PEG	1,2-Dimyristoyl-*rac-glycero*-3-methoxypolyethylene glycol
DMPC	1,2-Dimyristoyl-*sn-glycero*-3-phosphatidylcholine
DOPC	1,2-Dioleoyl-*sn-glycero*-3-phosphocholine
DOPE	1,2-Dioleoyl-*sn-glycero*-3-phosphoethanolamine
DOPG	1,2-Dioleoyl-*sn-glycero*-3-phosphoglycerol
DOTAP	1,2-Dioleoyl-3-trimethylammonium-propane
DOPS	1,2-Dioleoyl-*sn-glycero*-3-phospho-l-serine
DPHPG	1,2-Diphytanoyl-*sn-glycero*-3-phosphoglycerol
DPPC	1,2-Dipalmitoyl-*sn-glycero*-3-phosphocholine
DPPG	1,2-Dipalmitoyl-*sn-glycero*-3-phosphoglycerol
DSPC	1,2-Distearoyl-*sn-glycero*-3-phosphorylcholine
DSPE	1,2-Distearoyl-*sn-glycero*-3-phosphoethanolamine
HSPC	Hydrogenated soy l-phosphatidylcholine
MHPC	1-Myristoyl-2-hydroxy-*sn-glycero*-3-phosphocholine
POPC	1-Palmitoyl-2-oleoyl phosphatidylcholine
PEG	Polyethylene glycol

Lipid vesicles exhibit chemical, morphological and structural resemblance to cells, organelles, and extracellular vesicles by virtue of their compartmentalisation by a lipid membrane. The membrane scaffolds enable lipid vesicles to mimic cellular functionalities, allowing them to encapsulate biomolecules, maintain out-of-equilibrium conditions, and facilitate various biochemical reactions. They allow the buildup of concentration gradients, the maintenance of homeostasis, and the preservation of cell shape and structural integrity. Additionally, membranes control, which molecules pass in and out of the cell, serve as the basis for internal cellular organisation, and play a key role in inter- and intra-cellular communication. The generation of artificial lipid vesicles was first reported by Bangham *et al.*^5^ in 1964. Since then, they have been investigated within the fields of membrane biophysics,^6–10^ drug delivery,^11,12^ and synthetic biology.^13,14^

Generally, vesicles with diameters smaller than 100 nm are described as ‘small’ or ‘nano’, and vesicles with diameters larger than 1 μm are described as ‘giant’. Those with diameters between 100 nm and 1 μm are described as ‘large’. ‘Unilamellar’ means possessing only one lipid bilayer, and ‘multilamellar’ means possessing a several of lipid bilayers. Nano-sized lipid particles without a distinct lipid bilayer structure are defined as lipid nanoparticles (LNPs). Size and lamellarity are commonly referenced properties for classifying lipid vesicles, as each determines the relevant liposome applications to a considerable extent.^11,15^ For example, small unilamellar vesicles (SUVs) are widely used as drug carriers (*i.e.*, Doxil®^15^) and lipid nanoparticles are used to deliver nucleic acids (*i.e.*, COVID-19 mRNA vaccines^16^). Giant unilamellar vesicles (GUVs) are considered ideal platforms for engineering artificial cells, which aim to mimic the structures, functions, and behaviours of cellular systems.^17^

Vesicles can exhibit a range of features, with the significance of each feature varying based on their intended applications.^18^ When reconstituting membrane proteins into lipid vesicles, it is important to consider lipid composition, surface charge and bilayer asymmetry. If vesicles are engineered as artificial cells and bioreactors, high encapsulation efficiency and low polydispersity index (PDI, describing the size distribution of particles, defined as the ratio of the square of the standard deviation of particle size to the mean diameter) are important. When applied as carriers of drugs and nucleic acids, precise control of vesicle stability and cargo release are added to the list of key metrics. Whether in research or industry, the practical use of lipid vesicles requires robust production methods. When assessing the methods for preparation, engineering indexes, such as ease of application, reproducibility, and production rate must be taken into consideration.^19^ Additionally, the production of lipid vesicles typically involves dissolving lipids by organic solvents, thus the potential presence of residual organic solvents in the membrane must be factored in the further purification stages.^20^

### 1.2 Conventional methods for lipid vesicle preparation

Since lipid vesicles were first synthesised, a diverse repertoire of methods for the construction have been developed, most of which can be grouped into three main types.^21–23^

1) Mechanical dispersion methods: a lipid film is hydrated by an aqueous buffer into an uncontrolled vesicle dispersion.^21^ These polydisperse lipid vesicles are then homogenized by mechanical processing. Typically, vesicles can be generated by film hydration,^22^ electroformation,^24^ sonication,^25^ and further processed by membrane extrusion.^26^

2) Solvent dispersion methods: first, a water-in-oil emulsion is formed by mixing an organic solvent dissolving lipids with an aqueous solution. Next, the organic solvent is then removed, and the vesicles are formed spontaneously and simultaneously. Typical solvent dispersion methods include organic solvent injection^27^ and reverse-phase evaporation,^28–30^ emulsion phase transfer.^31–34^

3) Detergent depletion methods: lipids are dissolved in an aqueous solution containing detergent above this detergent's critical micelle concentration (CMC; the concentration of amphiphile above which self-assembly into mixed micelles occurs). As the detergents are removed through dialysis^35^ or dilution,^36^ the micelles become increasingly enriched in lipids and eventually form vesicles.

The methods mentioned above are not mutually exclusive but may be combined. For example, when preparing GUVs nesting small proteoliposomes,^37^ film hydration is typically followed by extrusion to achieve SUVs with uniform size distribution and lamellarity, detergent depletion can be conducted on extruded SUVs to reconstitute membrane proteins, and emulsion phase transfer can be used to encapsulate the small proteoliposomes into GUVs. Traditional methods are versatile and have been effective for decades in many applications. Extrusion is commonly employed for SUVs and LUVs,^26^ and methods such as electroformation^24^ and emulsion phase transfer^32–34^ are well established for GUVs. However, conventional methods are usually conducted in bulk with limited process control, and therefore suffer from limitations associated with batch-based production,^19^ poor reproducibility,^18^ large reagent consumption, and high waste.^19^ Many classical methods also often have poor control over membrane properties,^38^ show low encapsulation efficiency, and do not enable sufficient control over architecture, membrane asymmetry, sub-compartmentalisation and spatial organisation of compartments.

### 1.3 Microfluidics technologies and lipid vesicle preparation

Emerging microfluidic production of lipid vesicles could provide an effective solution to the issues of conventional bulk-based methods. Microfluidics can be defined as “the science and technology of systems that process or manipulate small (1 × 10^−9^ to ×10^−18^ L) amounts of fluids, using channels with dimensions of 10–100 μm”.^39^ The confined microenvironment where microfluidic procedures occur is characterized by low Reynold's number^15,18^ as laminar flows (Box 1).

Since the 1990s microfluidics has become a flourishing interdisciplinary field and has seen applications within both academic and commercial fields.^40^ A characteristic advantage of microfluidics is the size effect at the micron length scale which enables unique properties. For example, relatively small heat and mass transfer distances support fast reactions. In addition, the capillary effect becomes dominant owing to large surface-to-volume ratios, which can be advantageous in certain scenarios. The development of microfluidic devices has also benefited from advances in fabrication technologies, including soft lithography^41^ and dry etching.^42^ These advanced manufacturing technologies can yield intricate microstructures that enable sophisticated functions and enhanced device performance.^43^ A number of materials have been used for constructing microfluidic devices such as silicon polymers, glass, paper, thermoplastics, hydrogels, and thermosetting plastics.^40,43^ The materials most applied for lipid vesicle generation are glass and polydimethylsiloxane (PDMS). These materials provide high optical transparency, can be easily surface-modified, and are structurally rigid.^44^

**Box 1. Reynold's number**

Reynold's number (Re) is a dimensionless number calculated from the ratio of inertial to viscous forces. Re is often used to profile the flow regime within microfluidic devices.1
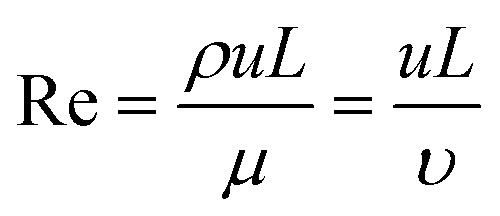
where *ρ* is the density of the fluid, *u* is the flow speed, *L* is a characteristic linear dimension, *μ* is the dynamic viscosity of the fluid, *υ* is the kinematic viscosity of the fluid.
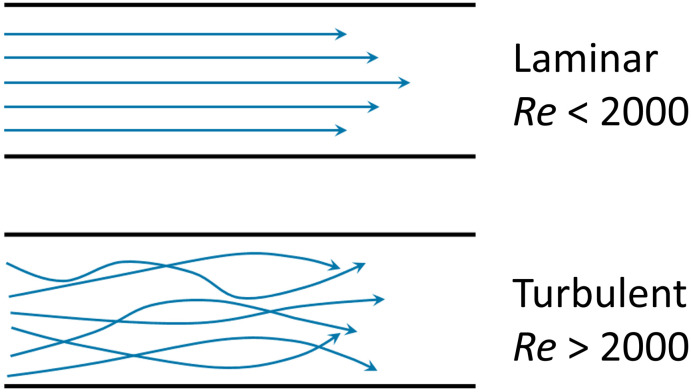

Box Fig. 1. Laminar flow and turbulent flow. The velocity profiles are portrayed in blue lines and the black lines represent channel walls.

At low Reynolds numbers (Re < 2000), fluid flow is dominated by laminar (sheet-like) flow. For multiple phases at low Reynold's number, the mixing process is governed by molecular diffusion and can be modelled using Fick's law. At high Reynold's number (Re > 2000) onset of turbulent flow is often observed. The multiphase mixing processes of turbulent flow are dominated by inertial forces and result in complex kinetics. Generally, laminar flow is more favourable for microfluidic vesicle preparation. The behaviour of laminar flow is more predictable and controllable than that of turbulent flow. Thus, the properties of resultant vesicles can be tuned in the laminar regime by adjusting parameters such as flow rate and chip geometry.

**Fig. 2:**
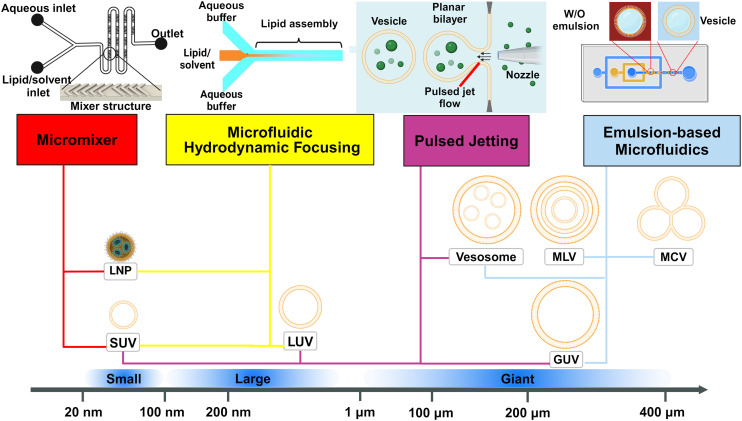
Schematic representation of the dominant microfluidic platforms for preparing various lipid vesicles. Vesicles with diameters smaller than 100 nm are described as ‘small’ or ‘nano’, this includes small unilamellar vesicles (SUVs) and lipid nanoparticles (LNPs). Vesicles with diameters between 100 nm and 1 μm are described as ‘large’. Vesicles with diameters larger than 1 μm are described as ‘giant’, including giant unilamellar vesicles (GUVs), vesosomes (vesicle-in-vesicle), multilamellar vesicles (MLVs) and multicompartmental vesicles (MCVs). Microfluidic platforms represented by micromixers (reproduced from ref. 45 with permission from the American Society of Gene & Cell Therapy, copyright [2012]) and MHF (reproduced from ref. 15 with permission from Springer Nature, copyright [2016]) have demonstrated great potential in preparing lipid vesicles with nanoscale sizes for medical applications. Emulsion-based microfluidics focuses on preparing giant liposomal products as cell models or bioreactors from water-in-oil (W/O) emulsions. The pulsed jetting method (reproduced from ref. 46 with permission from the American Chemical Society, copyright [2007]) can prepare vesicles of ‘small’, ‘large’, and ‘giant’ sizes.

The broad scope of microfluidic technologies is complemented by the “lab on a chip” concept.^18–20^ Currently, many examples of microfluidic platforms for liposome synthesis exist (Fig. 2). When compared with the conventional bulk liposome preparation methods, the emerging microfluidic methods have enabled control over both the preparation processes (*i.e.*, rate control) and the properties of liposome products (*i.e.*, size control).^15,18^ Microfluidic lipid vesicle preparation methods also enable continuous and high-throughput production, facilitate integration with on-chip manipulation and analysis and involve cost-effective fabrication.^18–20^

In this review, we describe and discuss principal microfluidic methods for synthesizing lipid-based nanocarriers and cell-sized lipid vesicles (Fig. 2). Microfluidic hydrodynamic focusing (MHF) and micromixers are highlighted as promising platforms for the large-scale production of lipid-based nanocarriers to deliver drugs, proteins or nucleic acids. Emulsion-based microfluidics is ideal for the continuous generation of cell-sized lipid vesicles, supporting user-defined compartmentalisation and membrane asymmetry. Pulsed jetting can produce vesicles of both nano and micro sizes. We also include on-chip hydration and on-chip electroformation in these two sections respectively, as they represent the microfluidic refinement of the classic methods.

## 2 Microfluidics for preparing lipid-based nanocarriers

### 2.1 Lipid-based nanocarriers

**Fig. 3:**
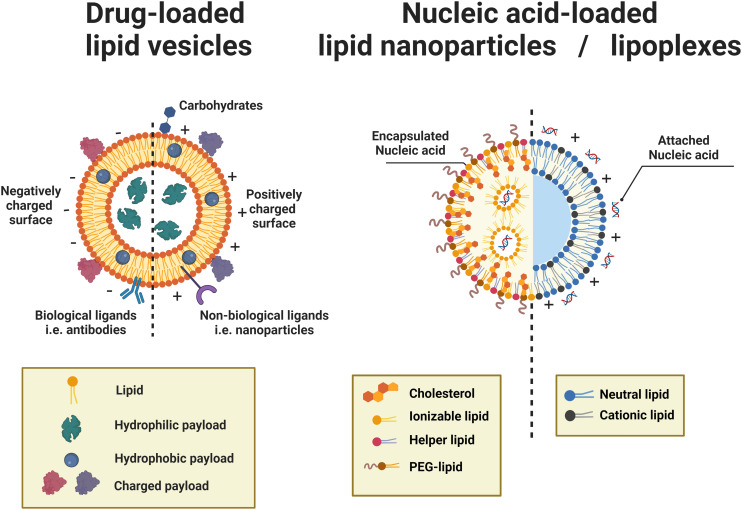
Schematic representations of drug-loaded lipid vesicles (left) and nucleic acid-loaded lipid nanoparticles (LNPs)/lipoplexes (right). For drug-loaded vesicles, different types of drug molecules can be loaded through different mechanisms. For active targeting and controlled release, ligands can be attached. The dashed line indicates that vesicle surfaces can be modified to be neutral, negative, or positive; not a mix of charges on the same vesicle. For the delivery of nucleic acids, LNPs (left half) are inverted micelles whose inner cores are occupied by cationic or ionizable lipids, which are usually formed by passive loading, while lipoplexes (right half) retain the continuous bilayer structure of their precursor liposomes, which are usually formed by active loading.

Typically, lipid-based architectures for medical applications require an average diameter smaller than 100 nm.^15^ The prospective nanoarchitectures can be simple small unilamellar vesicles (SUVs) or more complex lipid nanoparticles (LNP) or lipoplexes. The distinction between these is that generally, SUVs (or liposomes) are spherical vesicles with a lipid bilayer encapsulating an aqueous core, while lipid nanoparticles are solid or semi-solid particles primarily composed of lipid aggregates, often lacking a distinct bilayer structure. They have both been extensively used as nanocarriers to deliver drugs,^12,22^ imaging agents,^47^ genetic materials^48,49^ and vaccines^16,50^ (Fig. 3). Compared to delivering free drugs directly, encapsulating by lipid-based scaffolds protects the cargoes from clearance by the immune system and degradation driven by changes in pH or enzymatic attack, leading to longer circulation time and lifetime.^11,51^ The small size (<100 nm) contributes to longer blood circulation time due to their reduced uptake by the mononuclear phagocytic system (MPS).^52^ Generally, particles should be larger than 8 nm to avoid kidney clearance.^53^ The size and lamellarity affect both the efficiency of encapsulating cargo^54^ and the stability of nanocarriers,^55^ which varies as the lipophilicity of cargoes and lipid compositions are changed.

Lipid-based nanocarriers enable targeted delivery and controlled release by surface modification and composition alteration. Passive targeting effects, such as the enhanced permeability and retention (EPR) effect, accumulate drug-loaded lipid nanoparticles in tumour tissues.^56,57^ Active targeting can be achieved by attaching functional ligands, antibodies and carbohydrate moieties to the vesicle surface, which selectively bind to the specific receptors and antigens on the surface of the targeted cell.^12,58^ Stimuli-responsive liposomes facilitate a site-selective release manner responding to endogenous microenvironmental changes such as pH, enzyme and redox, or externally applied stimuli such as temperature, light and ultrasound.^59–61^

Lipid-based nanocarriers can be taken up by cells through several mechanisms, which often function in parallel.^22,62^ The major mechanism of cellular uptake of lipid-based nanocarriers is endocytosis, transferring the entire nanoparticle across the cell membrane and into the cell.^51,63^ In some cases (*i.e.*, non-bilayer phases such as cubosomes with a diameter of 150–300 nm), direct membrane fusion between the moiety of lipid carriers and the cellular membrane may take place.^51^ Compared to endocytosis, direct membrane fusion is relatively rare.^63^

For the drug-loaded lipid vesicles (Fig. 3 left), their structures are amenable for the encapsulation of both hydrophilic and hydrophobic cargoes. Hydrophilic cargoes are dissolved in the interior aqueous volume, while hydrophobic molecules are trapped within the lipid bilayer. Supramolecular charged payloads can be attached to the external surface of vesicles through electrostatic interactions. Furthermore, cargo can be loaded passively or actively.^64,65^ Passive encapsulation loads molecules of interest as the self-assembly of vesicles occurs. By contrast, active cargo loading, oftentimes achieved by the pH gradient method, drives cargo into preformed lipid vesicles. Generally, passive loading often has relatively low encapsulation efficiency while active loading can reach extremely high encapsulation efficiency.^64^

Specifically, lipid nanoparticles (LNPs) or lipoplexes (Fig. 3 right) are used to define the lipid-based nanoarchitectures that deliver nucleic acids.^48,49^ LNPs are typically inverted micelles whose inner cores are occupied by cationic or ionizable lipids.^66^ LNPs are often formed by the direct coassembly of lipids and nucleic acids, a format of passive loading.^48^ Conversely, lipoplexes are vesicle-like complexes formed by attaching nucleic acids to the surface of preformed liposomes.^48^ Owing to the active loading without destroying the preformed liposomes, lipoplexes retain the continuous bilayer structure of their precursor liposomes.^66^

The formulation of LNPs often involves positively charged lipids, helper lipids, cholesterol, and PEGylated lipids.^48^ Positively charged lipids, including permanently cationic or ionizable lipids, are essential for LNP synthesis as they condense and entrap negatively charged nucleic acids through electrostatic interactions.^49^ Particularly, ionizable lipids present positively charged at acidic pH (below the p*K*_a_) but switch to neutral when the pH is above the p*K*_a_. During formulation at acidic pH, protonated ionizable lipids allow high encapsulation efficiencies of nucleic acids by promoting electrostatic interactions. During storage and *in vivo* circulation where the physiological pH is above the p*K*_a_, neutral ionizable lipids support the stability of the lamellar phase and avoid nonspecific adsorption of negatively charged biomolecules, respectively. When LNPs reach endosomes, the acidic environment reprotonates ionizable lipids, which facilitates membrane fusion between the LNP and endosomal membrane, forming a non-bilayer hexagonal (H_II_) phase. The endosomal membrane is destabilized temporarily, and the payload within LNPs can escape the endosome into the cytosol of the cell.^48,49^ Some helper lipids, such as DOPE, help improve transfection efficiency by encouraging the formation of the H_II_ phase and facilitating membrane fusion, whilst some, like DSPC, improve particle stability by stabilizing the bilayer. The use of cholesterol and PEG lipids also enhances LNP stability. Cholesterol increases the overall structural integrity of the LNPs, and PEG lipids protect the LNP surface from opsonization, reticuloendothelial clearance, and destabilization during systemic circulation.^49,67^

Lipoplexes are often composed of cationic and neutral lipids (also called ‘helper lipids’ or ‘co-lipids’).^48^ Like in LNPs, in lipoplexes, cationic lipids interact with the nucleic acids, support stable storage, and facilitate cellular entry and subsequent cargo release, while neutral lipids help with formation-related phase changes and reduce interparticle aggregation. Different from the popular use of ionizable lipids in LNPs, most of the cationic lipids used in lipoplexes are permanently charged or only slightly ionizable.^48^

### 2.2 Microfluidic hydrodynamic focusing (MHF)

#### 2.2.1 Overview

**Fig. 4:**
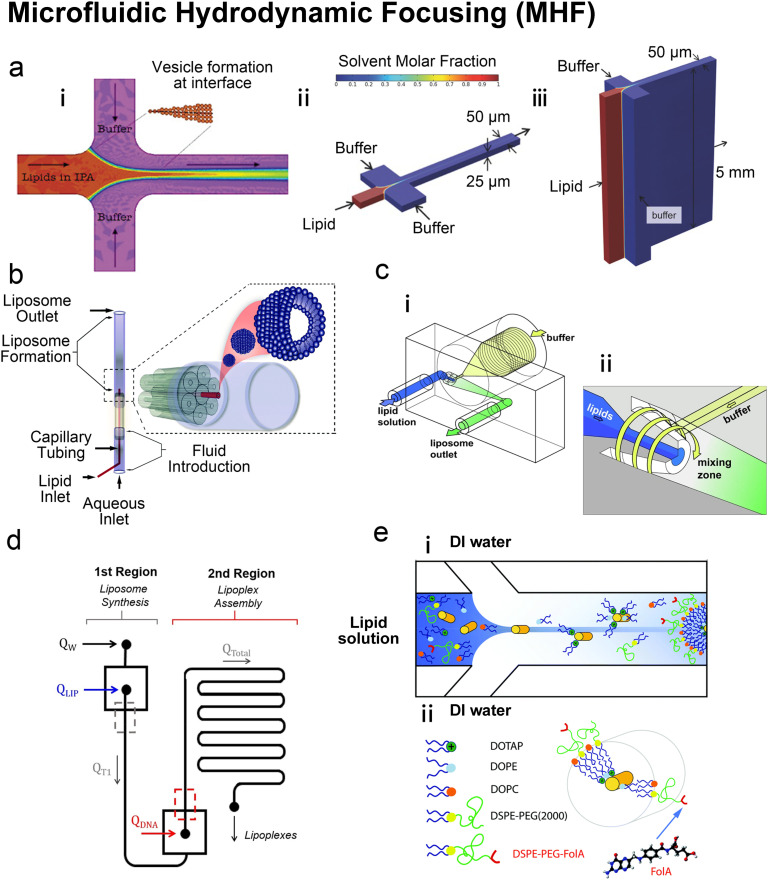
Microfluidic hydrodynamic focusing. a| (i) Schematic of liposome formation through microfluidic hydrodynamic focusing. Two aqueous streams focus one lipid organic stream. Reproduced from ref. 68 with permission from the American Chemical Society, copyright [2004]. Numerical simulations comparing ethanol concentration profiles within MHF (ii) and VFF (iii) systems. In the VFF system (not to scale), its microchannel aspect ratio is 1000 : 1, much larger than 0.5 : 1 in the conventional MHF system. Reproduced from ref. 71 with permission from John Wiley and Sons, copyright [2015]. b| Schematic of capillary focusing liposome formation device (not to scale). A lipid alcohol solution is continuously injected into the intra-annular capillary tubing and hydrodynamically focused in three dimensions by an exterior sheath flow of aqueous buffer from a surrounding glass multi-capillary array. Reproduced from ref. 72 with permission from the Royal Society of Chemistry, copyright [2014]. c| Microfluidic vortex focusing (MVF) device design and operation. (i) The MVF device design consists of two inlets conjoining at the annular junction, a conical mixing region, and an outlet. (ii) Magnified view on the annular junction. Mixing is improved through vortex focusing. Reproduced from ref. 73 with permission from Springer Nature, copyright [2022]. d| Schematic representation of the microfluidic devices for a two-stage formation of cationic liposome at the 1st MHF region and pDNA loaded lipoplexes at the 2nd MHF region. Reproduced from ref. 74 with permission from Elsevier, copyright [2017]. e| The assembly (i) and structure (ii) of mNALPs in a microfluidic T-junction chip. Mixing of lipid solution and DI water at the nanolitre scale in microfluidic channels leads to rapid changes in solvent properties that drive particle formation. Reproduced from ref. 75 with permission from the Royal Society of Chemistry, copyright [2017].

Microfluidic hydrodynamic focusing (MHF) is a nanoparticle preparation method centred around mixing miscible solvents. Jahn *et al.* laid the groundwork for the MHF method for vesicle synthesis.^68–70^ In a typical flow-focusing MHF chip (Fig. 4a (i)), an organic solution containing the desired lipids is injected from the central channel. This organic stream is focused by two lateral aqueous streams from side channels.^68^ The organic solvent must both solvate lipids and be miscible with the aqueous buffer of which isopropanol and ethanol are the most common choices. As the streams mix in a controlled manner (often *via* diffusion), the lipids start to self-assemble when transitioning from an organic solvent (where lipids are miscible) to an aqueous environment (where lipids are immiscible). The highly controlled and uniform mixing leads to efficient vesicle formation and allows precise control over vesicle size and lamellarity.

Based on the well-known non-equilibrium model put forward by Lasic^76^ about vesicle formation, Jahn *et al.*^70^ hypothesised that the formation of vesicles in MHF is kinetically controlled. The properties of vesicles, especially the size, depend on the formation, growth and closure of the intermediates, which are disc-like fragments or oblate micelles.^76^ In MHF, the diffusion and convection of solvent molecules lead to a spatial and temporal gradient of polarity in the surrounding fluidic environment of the amphiphilic molecules. When the concentration of the organic nonpolar solvent decreases to a critical concentration, the self-assembly of intermediates is triggered at the alcohol–water interface.^15,70^ As these intermediates grow, their transportation by axial advection dominates as the diffusion coefficient decreases due to the decline in the lipid concentration gradient. Consequently, the increasing polarity of their surrounding environments triggers the rearrangement of the micellar disc intermediates into lipid vesicles by the hydrophobic effect. The existence of the disc-like intermediate assemblies was proved by rapidly freezing the MHF chip and observation under cryo-scanning electron microscopy.^77^ Zook *et al.*^78^ hypothesised that the growth of intermediates in MHF should be approximately proportional to the ratio of the membrane bending elasticity modulus to the line tension of the hydrophobic edges of the lipid bilayer disc. Based on this hypothesis, they successfully predicted the effects of temperature, acyl chain length of lipids, and flow rate conditions on vesicle sizes. Choi *et al.* synthesized bilayer micelles or so-called bicelles through hydrodynamic focusing. Bicelle has a discoidal shape with a bilayer domain composed of long-chain lipids and a single-layer rim composed of short-chain lipids.^79^ Choi *et al.* verified that the transition from bicelles to vesicles could be achieved through dilution, with the size of vesicles controlled by lipid composition, mixing time, and temperature. Apart from producing lamellar vesicles, Pilkington *et al.* reported the use of MHF in generating high-order lipid assemblies with non-lamellar phases.^80^ Lyotropic liquid crystalline (LLC) nanoparticles (cubosomes and hexosomes) were produced rapidly and continuously with tunable sizes controlled by flow rate ratio (FRR).

The size tunability by FRR in MHF is attributed to the controllable length of its growth phase.^70^ In MHF, the ratio between radial diffusion speed and axial convection speed depends on the FRR between the outer aqueous flows and the central organic flow (Box 2). With increasing FRR, the advective transportation of intermediates is faster which reduces the growth phase and results in smaller vesicles. With FRR increased to a limit, the decrease of diameter stops, where the limit is determined by the intrinsic geometry of the microfluidic device used.^69^

TFR represents the total sum of aqueous and alcohol flow rates. It relates to the residence time within the microfluidic device and the liposomal production rate. The effect of TFR on diameter is currently controversial. Carugo *et al.*^15^ reported that TFR had no significant impact on resultant particle diameter. Jahn *et al.*^70^ reported that the average size of liposomes increased with the TFR when the FRR was fixed and relatively small (*i.e.*, FRR = 14) while the size of liposomes became independent of TRF at high focusing conditions (*i.e.*, FRR = 49).

**Box 2. Illustration of MHF mechanism**

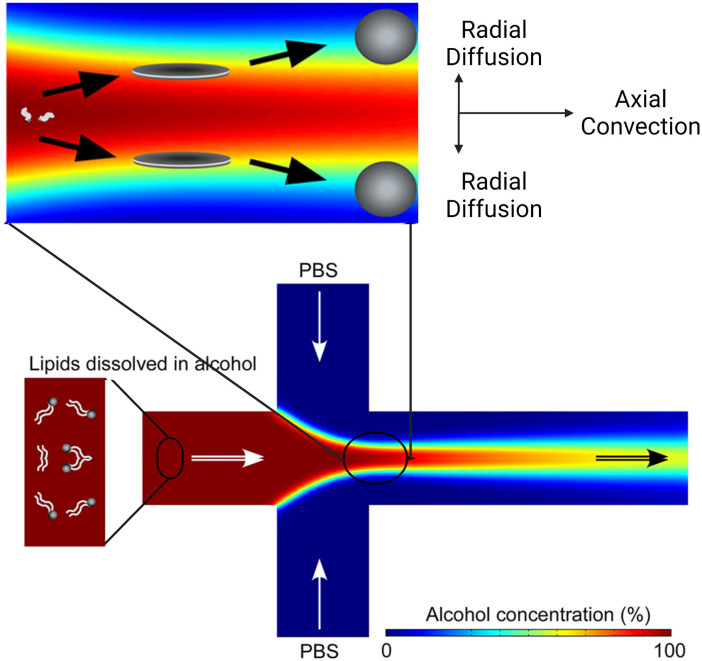
Box Fig. 2. Simulation of the MHF process. Radial diffusion and axial convection happen in the microfluidic channel. As the environmental polarity changes, lipids assemble into disc-like intermediates and form vesicles at critical alcohol concentration. Reproduced from ref. 77 with permission from the American Chemical Society, copyright [2013].2
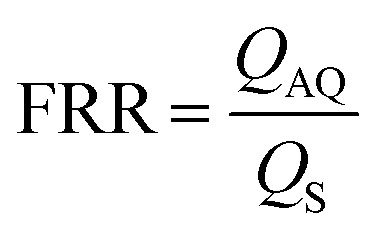

3 TFR = *Q*_AQ_ + *Q*_S_

where the *Q*_AQ_ is the total volumetric flow rate of the aqueous stream(s) and the *Q*_S_ is the total volumetric flow rate of the organic solvent streams.

#### 2.2.2 Methods

Jahn *et al.*^68–70^ initially applied DMPC and cholesterol as substrate lipids (DMPC : cholesterol : dihexadecyl phosphate (DCP) molar ratio = 5 : 4 : 1). Since the work by Jahn *et al.*, numerous lipid formulas have been investigated. PC formulas such as soy PC,^15^ POPC^81^ and DPPC^82^ were employed. Choi *et al.*^79^ tested four PC lipids with different transition temperatures. Long-chain lipids dissolved in IPA such as DMPC, POPC or DPPC were mixed with short-chain DHPC dissolved in PBS, and bicelles and vesicles were formed under different conditions. Carugo *et al.*^15^ and Amrani *et al.*^82^ investigated the effects of charged lipids, such as DOPG^82^ and DDAB.^15,82^ They found increasing liposome sizes with increasing quantities of charged lipids. Cationic lipids were added to the lipid formula for the delivery of nucleic acids,^74,75,83,84^ and PEG lipids were added for smaller particle sizes and higher stability.^71,72,75,84,85^

Beyond the lipid formula, MHF Chips with different geometries,^15,69,70,86^ channel dimensions,^71^ and device materials^72,73^ have been implemented. Notable adaptations were conducted by the group of Hood and DeVoe.^71–73^ They updated the previous planar MHF chips into 3D versions for reduced polydispersity indexes and improved production rates. This group first substituted the PDMS channels of Jahn *et al.*'s MHF chips^68^ with concentric capillary arrays (Fig. 4b). In this capillary system, a super large FRR of 5000 was successfully applied, and SUVs with diameters ranging from 106 nm to 140 nm were produced at TFR = 5 mL min^−1^.^72^ They also developed the vertical flow focusing (VFF, Fig. 4a (iii)) approach by greatly increasing the aspect ratio of MHF chips, which resulted in wide and thin liquid sheets for mixing.^71^ Compared with previous planar MHF^68–70^ and the capillary system,^72^ the production rate of VFF (95 mg h^−1^ lipid) was improved by nearly two orders of magnitude and over an order of magnitude respectively. Recently, Han and DeVoe *et al.* further updated their capillary system by setting the steam of aqueous buffer perpendicular to the lipid alcohol stream^73^ (Fig. 4c). A highly vortical flow was established around the lipid stream to sheath it for flow focusing and generate a vortex for the promotion of mixing. PEGylated liposomes as small as 20 nm could be formed at a mass production rate of over 20 g lipid per h. Carugo *et al.*^15^ designed several MHF microdevices for industrial liposome production, which supported FRR ranging from 5 to 100 and TFR ranging from 3–18 mL min^−1^. Their products presented comparable qualities to those produced by laboratory MHF devices.

SUVs prepared by MHF have demonstrated great potential as drug carriers. Lin *et al.*^87^ conducted a systematic characterization of passive drug loading by MHF, using fluorescent substances to simulate hydrophilic drugs and hydrophobic drugs. Either loaded separately or concurrently, the encapsulation efficiencies of both types of drugs were improved as the FRR increased from 10 to 50. The encapsulation efficiency of the hydrophilic model drugs reached around 90% at FRR = 50 although that of the hydrophobic model drugs only reached 25% at the same FRR. Empty SUVs and hydrophilic drug-loaded SUVs had similar sizes, whilst loading hydrophobic drug simulants led to larger vesicle sizes. Pilkington *et al.* encapsulated curcumin (hydrophobic) and carboxyfluorescein (hydrophilic) in their MHF-generated hexosomes and cubosomes.^80^ Curcumin and carboxyfluorescein loading efficiencies for monoolein-based cubosomes and phytantriol-based hexosomes were all around 50%. Phytantriol cubosomes had lower loading efficiencies, with curcumin at around 40% and carboxyfluorescein at 10%. The phytantriol cubosomes presented size-dependent fusogenic behaviour when delivering calcein (a self-quenching fluorescent dye) into GUVs. In a more recent work, Pilkington *et al.* applied MHF in synthesising nanosized liposome-in-liposome, which was termed as concentrisome.^88^ They introduced lipids through both lipid-containing ethanol solution and lipid-vesicle-containing aqueous solution. These pre-formed vesicles were covered by a second bilayer through an MHF process. The compartment between the inner and outer bilayers was supported and dimensionally controlled by the click-chemistry reaction between dibenzocylooctyl-lipids on the inner bilayer and azido-lipids on the outer bilayer. The improved architecture complexity allowed separate encapsulation of different cargo and multi-stage release triggered by different stimuli.

Balbino *et al.* prepared cationic liposomes with a mixture of egg PC, DOPE and DOTAP (50 : 25 : 25 mol%) by MHF.^74,83^ The cationic liposomes formed by MHF were initially loaded with plasmid DNA (pDNA) using a batch mixing protocol.^83^ In a later trial, lipoplexes were assembled on a coupled MHF device^74^ where a formation of cationic liposomes through MHF was followed by an on-chip MHF attachment of pDNA (Fig. 4d). Compared with lipoplexes produced by the conventional extrusion method, the pDNA lipoplexes produced by MHF performed similarly in cytotoxicity and transfection when treating human cervical cancer (HeLa) and prostate cancer PC3 cells *in vitro*.^74^

Koh *et al.* designed a 5-inlet MHF device and prepared multilamellar lipid nanoparticles with Bcl-2 antisense oligodeoxyoligonucleotide (ODN) encapsulated.^84^ In their setup, a protamine/lipid central ethanol stream was focused by two ODN buffer streams at the first junction, which were subsequently focused by two more protamine/lipid ethanol streams. Their products consisted of ODN : protamine : lipids (1 : 0.3 : 12.5 wt/wt ratio) and the lipids contained DC-Chol : egg PC : DSPE-PEG (40 : 58 : 2 mol%). Samples collected from the chip were then dialysed to reduce residual ethanol and the unbound ODN, and partially neutralise the cationic DC-Chol moiety. After dialysis, the average particle size significantly reduced from 282.8 ± 24.0 nm to 106.8 ± 5.5 nm. Transferrin was incorporated as a targeting molecule for transferrin-positive K562 cells. Compared with bulk preparation, MHF presented comparable ODN encapsulation efficiency (71.3% ± 3.2% for bulk and 74.8% ± 3.8% for MHF) whilst the transferrin-targeted lipoplexes prepared by MHF down-regulated the Bcl-2 protein level more efficiently.

Krzysztoń *et al.* mixed lipids (DOPC : DOPE : DOTAP = 6 : 5 : 1 with extra 10 mol% of DSPE-PEG (2000) or DSPE-PEG(2000)-FolA) with double-stranded DNA or small interfering RNA in an isopropanol water mixture (IPA : H_2_O = 50 : 50).^75^ They diluted this mixture solution by 10 folds with deionized water on an MHF chip (Fig. 4e). The dilution through MHF yielded monomolecular nucleic acid/lipid particles (mNALPs) with small sizes (radius <50 nm). The mNALPS produced by MHF presented lower PDI compared with those produced by bulk vortex dilution. The encapsulation efficiency of nucleic acids was 20% higher using MHF than bulk vortex dilution. The mNALPs functionalized by folate exhibited high stability in blood serum and plasma. They were successfully targeted to folate-receptor-expressing epithelial cancer KB cells and demonstrated the potential in delivering siRNA into the cytoplasm. However, to compensate for the dilution effect, mNALP samples required further concentrating, which caused ∼30% material losses.

Kim *et al.* reported a single-step reconstitution method based on a co-flow MHF chip. Formation of high-density lipoprotein (HDL), encapsulation of hydrophobic molecules, and incorporation of functional nanocrystals were completed instantaneously and almost simultaneously.^89^ In biological systems, HDLs deliver native nucleic acids (*i.e.*, microRNA) to target cells *via* binding of apolipoprotein A-I (ApoA-I) to specific scavenger receptors on the membrane of target cells. HDLs also play critical roles in transporting cholesterol, signal lipids proteins and other biomolecules.^48^ Using DMPC and MHPC, HDLs synthesised by MHF were compared with those by the conventional incubation method. The microfluidic-synthesized HDLs had a diameter as small as 8.1 nm after purification and yielded 57 ± 11% ApoA-I. The yield of ApoA-I was slightly lower than the incubation method (59 ± 6%), but the synthesis time was greatly reduced from 16 hours to several minutes. Two hydrophobic molecules presented good encapsulation efficiency (94.2 ± 9.6% for 3,3′-dioctadecyloxacarbocyanine perchlorate (DiO) and 70.1 ± 7.0% for simvastatin) and maintained their functions as a fluorescent dye and anti-inflammatory drug respectively. Inorganic nanoparticles were also incorporated and functioned properly as imaging agents (Au for computed tomography, FeO for magnetic resonance imaging and quantum dots for fluorescence).

External electric fields were integrated with MHF platforms to produce liposomes by Modarres *et al.*^90^ AC electroosmosis was applied to generate phase-controlled mixing on an MHF chip, where the phase relation leading to the best mixing was strongly dependent on electrode orientation and biasing layouts.^90^ As the mixing efficiency was enhanced, better size distribution and higher concentrations of particles were achieved.

Finally, the application of MHF has also extended to assemblies of other organic polymers,^91–94^ inorganic nanoparticles,^95,96^ and a hybrid mixture of lipids and polymers.^85^ For cheaper and easier fabrication, the fabrication of MHF devices has also already extended from soft lithography^68^ to multilayer thermoplastic fabrication,^71^ 3D printing^97,98^ and microfluidic fibre wet spinning.^99^

#### 2.2.3 Summary and scope (Table 2)

Despite the advantages of MHF in preparing lipid vesicles and lipid-based nanocarriers, there are also some limitations. First of all, most MHF-involved publications use alcohol to dissolve lipids, but limited investigation has been made into the effects of residual alcohol in MHF products. In early reports, isopropanol (IPA) was the main solvent^68–70,75,77–79,81,87^ and was later replaced by ethanol,^15,71–74,82–84,86^ as ethanol is less toxic and complies with routine industrial processes.^15^ Dialysis can be used to remove alcohol,^84^ and residual ethanol up to 0.5% (v/v) is accepted under the guidelines in Ph. Eur. and USP. MHF inherently exhibits a strong dilution effect. Particularly, preparing smaller liposomes requires a larger FRR. For instance, liposomes with diameters smaller than 50 nm require FRR larger than 30, which means at least a 30-fold dilution.^70^ To prevent the final lipid concentration from being too low for clinical use, injection of high concentrations of lipids or postprocessing of concentrating, such as ultrafiltration,^87^ is usually necessary. However, increasing the initial injected lipid concentration can cause lipid precipitation at the focusing region^83^ and batch post-concentrating may result in mass loss.^74^ Secondly, in the aspect of encapsulation, molecules bound to the outer surface rather than trapped in the vesicles may lead to the overestimation of encapsulation efficiency.^100^ To evaluate the overestimation caused by external binding, empty liposomes can be used as the control group to gently mix with the molecules to be loaded by incubation.^100^ Depending on the interaction strength between the membrane and the externally bound molecules, dialysis or column chromatography may also remove the externally bound molecules to a certain extent. However, this will add several batch steps and increase the preparation time. Thirdly, while many studies exist focusing on optimizing the MHF vesicle products themselves, only a few reports have revealed improved production rates.^71–73^ Finally, liposomes or lipid-based nanoparticles produced by MHF are promising nanocarriers for drugs and nucleic acids but their potential in constructing membrane models for biophysical research use, such as membrane protein reconstitution, still needs further exploration.

**Table 2:** Summary of microfluidic hydrodynamic focusing (MHF)

Products	Empty SUVs/LUVs:^68–72,77,78,81,86,90^
	Bicelles:^79^
	Drug loaded SUVs/LUVs:^15,87^
	LNP:^75,82,84^
	Cationic liposomes:^83^
	Lipoplex:^74^
	High-density lipoprotein:^89^
	Cubosomes and hexosomes:^80^
	Liposome in liposome:^88^
Cargoes	Ivermectin:^15^
	Hydrophilic and hydrophobic drug simulants:^80,87–89^
	Peptides:^82,84^
	siRNA:^75,82^
	pDNA:^74,83^
	Protein:^89^
	Imaging agents:^89^
Chip materials	PDMS/glass:^15,68–70,74,79–84,87–90^
	Glass capillaries:^72,86^
	Cyclic olefin copolymer:^71^
	PEEK capillaries and stainless-steel mixer:^77^
	Glass wafer and Si wafer:^78^
Lipid compositions	PC lipid:^15,79,81,82,89^
	PC lipid & cholesterol & DCP:^68–71,73,78,86,87,90^
	PC lipid & cholesterol & PEG lipid:^71,72,88^
	PC lipid & charged lipids:^15,82^
	PC lipid & cationic lipid & PEG lipid:^84^
	PC lipid & DOPE & DOTAP:^74,83^
	PC lipid & DOPE & DOTAP & PEG lipid:^75^
	Monoolein, phytantriol, tocopherol acetate:^80^
Alcohol phase	IPA:^68–70,75,77–79,81,87^
	Ethanol:^15,71–74,80,82–84,86,88^
	Ethanol, methanol, chloroform:^89,90^

### 2.3 Micromixers

#### 2.3.1 Overview

MHF generally uses a large FRR to produce lipid-based nanocarriers, which results into a relatively low production rate due to the dilution effect. To overcome this limitation, novel types of micromixers are developed to form nanoscale vesicles under lower FRR.^101^ Micromixers are often used to describe devices with submillimetre length dimensions.^102^ Note that MHF can be seen as a type of micromixer in a broad sense, which relies on molecular diffusion to drive mass transportation and lipid self-assembly. In this part, we highlight micromixers that use chaotic advection and Dean vortices as the driving forces for mass mixing and lipid vesicle formation.

#### 2.3.2 Chaotic advection

Chaotic advection is the complex behaviour that a passive scalar, such as the concentration of a tracing particle, can attain, driven by the Lagrangian dynamics of the flow.^104^ In micromixers driven by chaotic advection, the stretching and folding of fluids make particles diverge exponentially and massively enhance mixing.^105^ However, although named ‘chaotic’, the chaotic advection is still laminar.^102^

The staggered herringbone mixer (SHM, Box 3), developed by Stroock *et al.*,^106^ was the first type of chaotic advection-based micromixer used for lipid vesicles and lipid-based nanocarriers preparation.^45,103,107–116^ On a typical SHM device, herringbone structures are placed on the floor of the microchannels to generate steady chaotic flows. These patterns of grooves on the floor create transverse flows that stretch and fold fluids over the cross-section of the channel, which enhances mixing and leads to reduced mixing length.

**Box 3. Illustration of chaotic advection in the staggered herringbone mixer (SHM)**

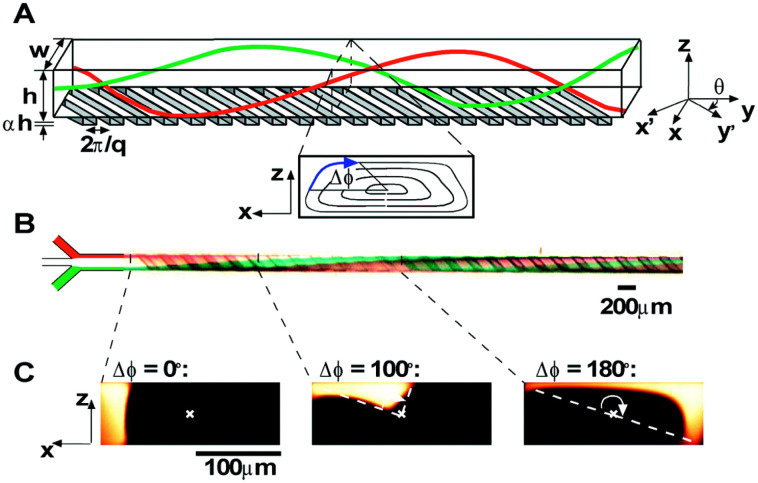
Box Fig. 3. Three-dimensional twisting flow in a channel with obliquely oriented ridges on one wall. Reproduced from ref. 103 with permission from the American Chemical Society, copyright [2012]. (A) Schematic diagram of a channel with ridges. (B) Optical micrograph showing a top view of a red stream and a green stream flowing on either side of a clear stream in a channel. (C) Fluorescent confocal micrographs of vertical cross sections of a microchannel.

Zhigaltsev *et al.* initiated the application of SHM in preparing ultra-small liposomes.^103,107^ In the earlier work,^103^ they mixed an ethanol stream containing lipids (POPC, POPC/cholesterol) with an aqueous steam on SHM. With FRR ≥ 3, bilayer vesicles of limited size (20–50 nm diameter) were formed. When dissolving triolein together with POPC in the ethanol stream. They achieved emulsions consisting of a triolein core and a POPC monolayer. The ammonium sulfate-based pH gradient method was applied to actively load doxorubicin into the liposomes and achieved approximately 100% encapsulation efficiency when the drug-to-lipid ratios were below 0.2 (molar ratio). Maeki *et al.* conducted parametric studies and mechanism analysis on the properties of empty POPC liposomes formed by SHM devices.^108,109^ In addition to the flow rate conditions, the SHM cycle numbers and the position of the first SHM were found to significantly affect the formation of small-size liposomes.^108^ The rapid decrease of the ethanol concentration around the disc-like intermediates was believed to be the reason why the products have small sizes. Chaotic advection in SHMs promoted mixing and reduced the residence time of intermediates at the critical ethanol concentration, which was estimated to be 60-80% ethanol for LNP formation.^109^ By regulating the residence time at the critical ethanol concentration, size tuning of LNPs at 10 nm intervals was achieved.^109^

In a later work published by Zhigaltsev *et al.*,^107^ more complex lipid compositions were investigated. An optimal formula composed of POPC, DPPC, cholesterol and DSPE-PEG2000 was identified, whose vesicular products had a diameter of 33 nm and exhibited adequate, stable drug retention when loading doxorubicin. The use of DPPC resulted in an improved retention profile in *in vivo* release studies. However, long saturated PCs (DPPC, HSPC) could not totally substitute POPC in this SHM-based method, of which the products aggregated and fused quickly under room temperature.^107^ Similarly, Cheung *et al.* loaded Doxorubicin into SHM-formed liposomes using the pH-gradient active loading method and achieved 80% encapsulation efficiency.^110^

Shah *et al.*^111^ compared SHM and extrusion for scale-up purposes. Liposomes composed of Egg sphingomyelin and cholesterol were prepared by these two methods. Water-soluble cargo vinblastine-*N*-oxide (CPD100) was encapsulated into the two types of pre-made empty vesicles by the A23187 (ionophore)-based pH gradient method. The CPD100-loaded vesicles produced by SHM exhibited identical physical and pharmacokinetic properties when compared to the extruded liposomes. Joshi *et al.*^112^ tested a passive drug loading approach on SHM, by dissolving a hydrophilic drug (metformin) in the aqueous steam and dissolving a lipophilic drug (glipizide) together with lipids in the ethanol steam. It is not surprising that they achieved lower loading efficiency (20–25% for metformin and 40–42% for glipizide), relative to the active drug loading conducted by Zhigaltsev *et al.*^103,107^ The two drugs could be loaded either individually or in combination, and the co-loading was found to have no impact on loading efficiency but accelerate the release.

**Fig. 5:**
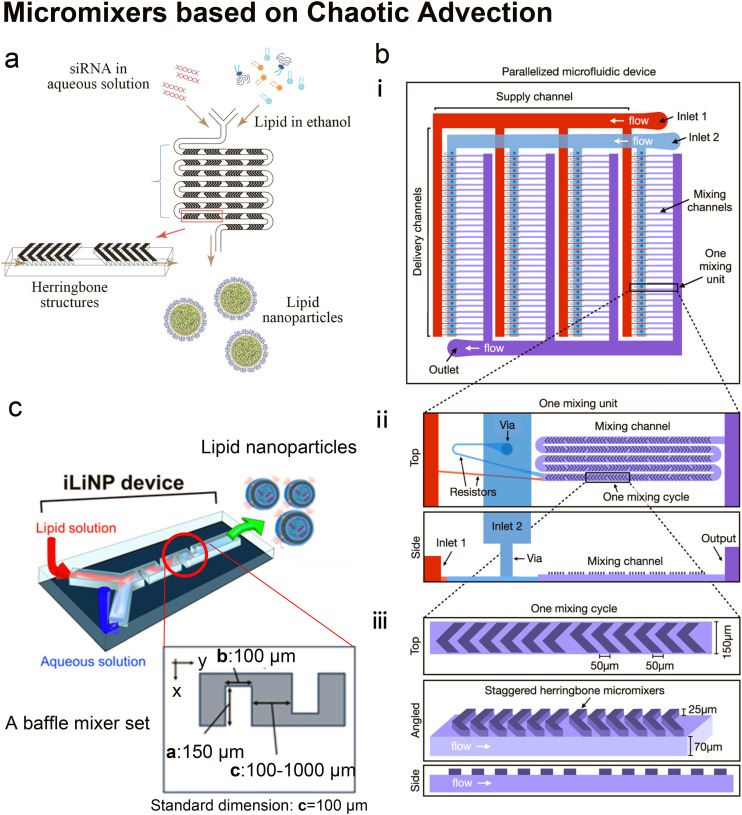
Micromixers based on chaotic advection. a| The schematic of lipid nanoparticle (LNP) small interfering RNA (siRNA) formulation strategy employing the staggered herringbone micromixer (SHM). Lipids in ethanol and siRNA in aqueous solution are pumped into the two inlets of the microfluidic device to produce lipid nanoparticles. Reproduced from ref. 45 with permission from the American Society of Gene & Cell Therapy, copyright [2012]. b| The schematic diagram for the design of a parallelized microfluidic device containing 4 rows of 32 mixing channels (i), highlighting the individual mixing unit design with a top view and a side view (ii) and the individual mixing cycle design with a top, angled, and side view (iii). The direction of flow is indicated by white arrows. Schematics are not to scale. Reproduced from ref. 114 with permission from the American Chemical Society, copyright [2021]. c| Three-dimensional and top views of the iLiNP device with the basic structure of 20 baffle mixer structure sets. Reproduced from ref. 119 with permission from the American Chemical Society, copyright [2018].

SHM's potential for loading genetic materials has also been investigated.^45,113,114^ Belliveau *et al.*^45^ pioneered the application of SHM in forming nucleic acid-loaded LNPs (Fig. 5a). Small interfering RNA (siRNA) was dissolved in an aqueous solution and mixed with an ethanol solution containing 40–60% ionizable cationic lipid (DLinKC2-DMA), helper lipid (DSPC), cholesterol and 1–5% PEG-lipids. LNPs had diameters ranging from 20 nm to 100 nm and polydispersity indexes as low as 0.02. Their optimized LNP siRNA systems achieved 50% target gene silencing in *in vivo* delivery tests, which was equal or superior to competitive products based on solvent dispersion method^117^ and extrusion method.^118^ Leung *et al.*^115^ systematically investigated the core structures of the siRNA-contained LNPs produced by the SHM micromixer. Their experimental results indicated that the interior lipid cores of LNPs contain siRNA duplexes complexed to cationic lipids, as well as phospholipid and cholesterol, and their modelling results described the cores as periodic structures of aqueous compartments, some of which had siRNA inside.

It is worth mentioning that Belliveau *et al.*'s idea of parallelization of SHMs to scale up LNP manufacturing^45^ was further developed by Shepherd *et al.*^114^ Shepherd *et al.* scaled up the throughput of SHM by incorporating 128 SHM mixing channels in one parallelized microfluidic device (PMD) and running 128 SHM mixing processes simultaneously^114^ (Fig. 5b). The ionizable lipid C12-200, a gold standard lipid for siRNA and mRNA delivery, was used as the main lipid component to produce LNPs. Factor V siRNA or luciferase-encoding mRNA in an aqueous phase was mixed with lipids in ethanol to induce self-assembly of the lipid nanoparticles. Compared with the single SHM device, this PMD increased production rates by over 100 folds, from mL h^−1^ up to L h^−1^ which is clinically relevant, and successfully preserved the desirable properties and functions of LNPs generated by single SHM. Compared with LNPs prepared by bulk mixing, the factor V siRNA LNPs and luciferase mRNA LNPs produced by PMD presented a 4-fold increase in hepatic gene silencing and a 5-fold increase in luciferase expression, respectively.

Kastner *et al.*^113^ prepared lipoplexes by incubating cationic liposomes (DOPE : DOTAP = 1 : 1 molar ratio) with plasmids containing luciferase genes in Opti-MEM. These cationic liposomes were previously prepared by SHM at FRR = 5 : 1, and had a diameter of 50–70 nm. The *in vitro* transfection efficacy of the lipoplexes was comparable to commercial Lipofectin™ and even higher at some optimal conditions. Their mathematical modelling confirmed that FRR impacts the liposome size, polydispersity index and transfection efficiency by the largest degree among the microfluidic parameters.

As predicted by Belliveau *et al.*,^45^ SHM has developed into a preferred method for the formulation of LNPs, due to its advantages of precise size control, high encapsulation efficiency, and improved scalability. SHM has also been commercialized by Precision Nanosystems, named NanoAssemblr Classic™, and widely used for research.^111–113^ Comparison between NanoAssemblr Classic™ and conventional hydration method in preparing lipoplexes was conducted by Elsana *et al.*^116^ The carboxymethyl-β-cyclodextrin was incorporated into cationic liposomes formed by DOTAP, DOPE and cholesterol (8 : 8 : 2 molar ratio). The formulations produced by NanoAssemblr Classic™ had smaller, more uniform sizes and more homogeneous zeta-potential as well as higher encapsulation efficiency when compared with those manufactured by the film hydration method.

Twisted channels have also been used to create chaotic advection.^119,120^ Kimura *et al.*^119^ designed a baffle mixer device named the invasive lipid nanoparticle production device (iLiNP, Fig. 5c), whose mixing rate was reported comparable to SHM. By changing the flow conditions and the baffle mixer dimensions, the size of LNPs could be precisely controlled at 10 nm intervals, ranging from 20 nm to 100 nm. On the iLiNP device, the factor VII siRNA was loaded by dissolving in the aqueous buffer and then mixed with an ethanol stream containing a pH-sensitive cationic lipid, cholesterol and PEG-DMG. The siRNA was delivered efficiently and showed good *in vivo* gene-silencing activity. In a recent work, the iLiNP device was used to deliver CRISPR/Cas ribonucleoprotein (RNP).^120^ With optimized device setting and lipid formulation, DNA cleavage activity and the aggregation of Cas enzymes were completely avoided. Gene disruption and base substitution reached 97% and 23% respectively *in vitro* without any apparent cytotoxicity. They also found that making the to-be-encapsulated RNPs more negatively charged by complexing single-stranded oligonucleotides greatly improved their delivery.

#### 2.3.3 Dean flows

Curved channels are fabricated in micromixers to create Dean flows for promoting mixing and generating liposomes with nano size.^101,121–129^ Dean flow is driven by lateral instability in curved channels (Box 4), and is characterized by the Dean number: De (eqn (4)). The Dean vortices are perpendicular to the main advection direction and rotating in opposite directions to each other, which enhances and accelerates the mixing process.^101^

**Box 4. Illustration of Dean flows**

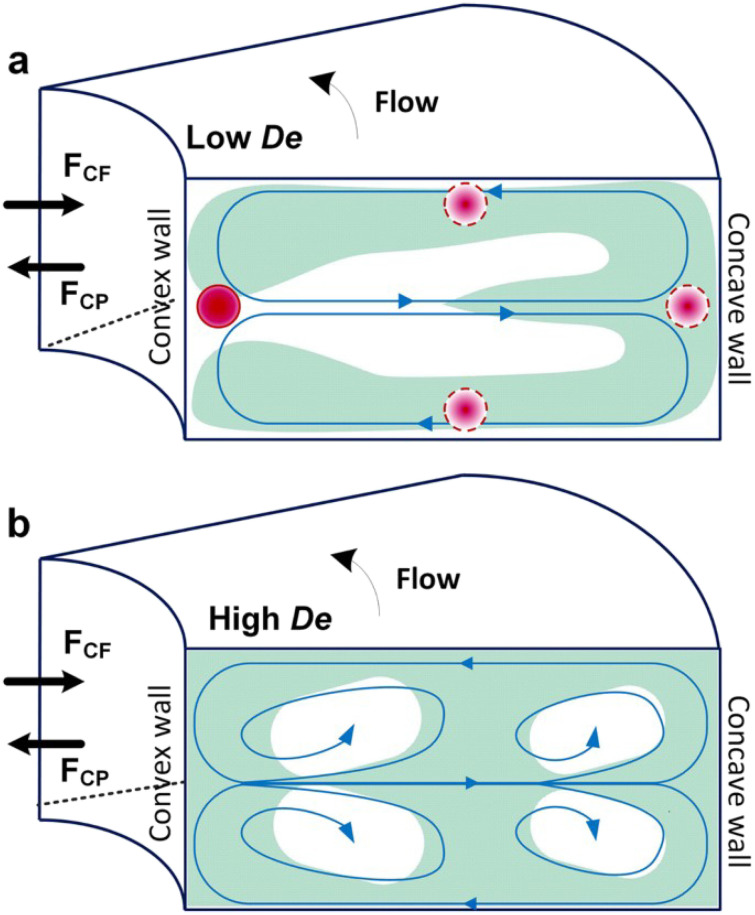
4
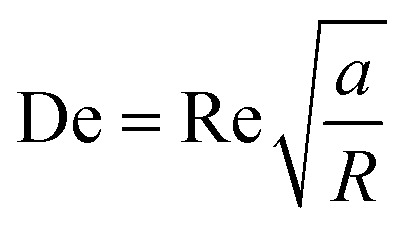
Box Fig. 4. Schematic illustrating Dean vortices. Where Re is the Reynolds number, *a* is the channel diameter and *R* is the radius of curvature. The Dean number represents the ratio between centrifugal force and inertial force. (a) When 10 ≤ De ≤ 150, the centrifugal force induces a secondary, transverse flow field characterized by two counter-rotating vortices in the upper and lower planes of symmetry of the channel. (b) When the De number is larger than 150, two additional vortices at the outer channel wall are formed.^121^ Reproduced from ref. 121 with permission from Springer Nature, copyright [2017].

**Fig. 6:**
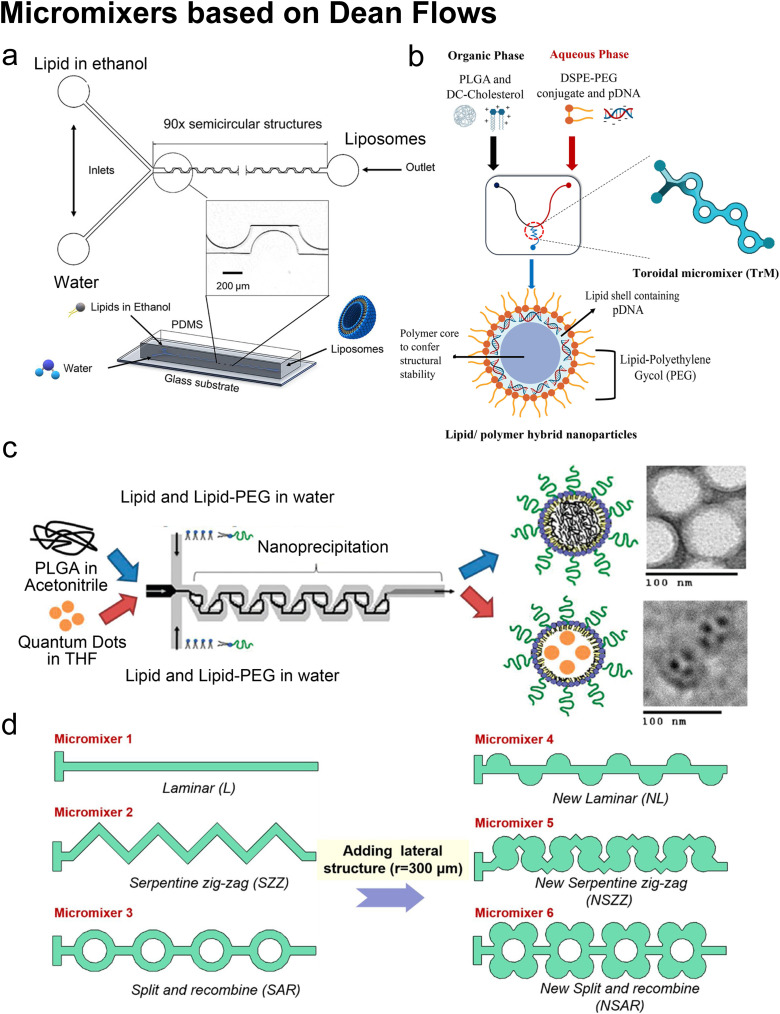
Micromixers based on Dean flows. a| Geometry and 3D model of a periodic disturbance micromixer (PDM). 90 semicircular structures were fabricated in the chip to generate Dean flows for mixing lipids in ethanol and water. Reproduced from ref. 101 with permission from the American Chemical Society, copyright [2021]. b| Lipid/polymer hybrid nanoparticle production using the toroidal micromixer (TrM). Reproduced from ref. 127 with permission from Elsevier, copyright [2022]. c| Nanoprecipitation of lipid-polymeric NPs in an MHF-SAR integrated device. Reproduced from ref. 128 with permission from the American Chemical Society, copyright [2010]. d| Applications of lateral structure to laminar, serpentine zig-zag and split and recombine micromixers, respectively. Reproduced from ref. 123 with permission from Elsevier, copyright [2020].

Lee *et al.* initialized using Dean flow in microfluidic devices to form nanoscale lipid vesicles.^122^ They designed a semi-circular contraction–expansion array (CEA) microchannel to create Dean flows. The induced Dean vortices led to 3D lamination by continuously splitting and redirecting fluid streams. The interfacial area between the IPA stream containing lipids and the PBS stream was increased due to the 3D lamination effect. This was believed to be pivotal for achieving small and monodisperse vesicles. Lee *et al.* found that the size of lipid vesicles was affected by both FRR and TFR, as they both affected the mixing efficiency. López *et al.* updated the CEA design by repeating the semicircle motif on alternating sides^101,126^ (Fig. 6a). They conducted a systematic study on parametric effects on the physicochemical properties of liposomes. FRR was found to have larger effects on liposome size and size dispersity, as compared with TFR. Liposome size was also affected by factors including temperature, lipid composition and concentration.

The toroidal mixer (TrM),^124,127^ also known as the ring mixer^125^ or split and recombine (SAR) mixer,^123^ is another typical Dean flow-based micromixer. Early involvement of lipids in SAR mixing was conducted by Valencia *et al.*^128^ (Fig. 6c). Following an MHF mixing region where lipids and PEG lipids were dissolved in water and mixed with a solution of poly-(lactic-*co*-glycolic) acid (PLGA) in acetonitrile, SAR mixing circles were set for nano-precipitation. Nanoparticles composed of a PGLA hydrophobic core, a PEG hydrophilic shell, and a lipid monolayer between the core and the shell were formed. These nanoparticles presented a narrow size distribution. They used the same setup and replaced PLGA in acetonitrile with quantum dots in tetrahydrofuran, by which the lipid-quantum dot nanoparticles were synthesised in a single step. The diameter (35 to 180 nm) and *ζ* potential (−10 to +20 mV in PBS, used to characterize a nanoparticle's surface charge), could be tuned by adjusting the composition and concentration of precursors.

An updated study was conducted on a commercial Y-shape TrM platform (NxGen Cartridge chip from Precision Nanosystems, Vancouver, Canada, Fig. 6b) by Santhanes *et al.*^127^ The cationic lipids (DC-cholesterol) and PLGA were dissolved in the organic phase, and DSPE-PEG2000 and pDNA were introduced through the aqueous phase. Lipid/polymer hybrid nanoparticles with a diameter of 100–120 nm were formed and presented 65% pDNA encapsulation efficiency as well as 20% transfection efficiency. Also using the NxGen, Ripoll *et al.* proposed optimal flow conditions for producing LNPs: large TFR (TFR > 4 mL min^−1^), long device (30 times the transverse dimension) and optimal FRR (FRR = 3, too large FRR would generate waste due to high dilution, too small FRR could not maintain required medium polarity).^125^

For comparing the NanoAssemblr Classic™ based on SHM and the NxGen based on Trm, the group of Perrie did systematic comparisons on the performance of SHM and TrM in producing drug/protein-loaded liposomes^124^ and nucleic acid loaded lipid nanoparticles.^129^ Polyadenylic acid,^124,129^ single-stranded deoxyribonucleic acid,^129^ messenger RNA^129^ and ovalbumin protein^124^ were passively loaded by being dissolved in the aqueous phase and mixing with the lipid-contained organic phase. Doxorubicin was actively loaded in the liposomes which were previously formed by the micromixers using a transmembrane pH gradient.^124^ Compared with SHM, TrM has similar performance in products' characteristics and parametric effects but supports higher production throughput, improving the production rate of NxGen to the good manufacturing practice (GMP) scale (20 L h^−1^).^124^

#### 2.3.4 Summary and scope (Table 3)

Micromixers are promising platforms for the production of lipid-based nanocarriers, especially those with diameters smaller than 100 nm. Taking advantage of promoted mixing under chaotic advection and Dean flows, the use of commercial devices such as Nanoassemmblr^101,112,113,116,124,129^ and NxGen^124,125,127,129^ has been in practice. Compared with MHF devices, lower FRR and higher TFR have been utilised in these micromixers. Lower FRR and higher TFR lead to higher lipid concentrations in the products and enhanced production rates, respectively. Commercialized from TrM, NxGen has enabled 200 mL min^−1^ TFR and 98% mRNA encapsulation efficiency.^130^ However, accompanied by lower FRR is a higher percentage of organic solvent in the product. If FRR is set to 3, there will be 25% vol ethanol left in the product. It is still an open question how much residual ethanol the liposomes produced by micromixers possess after dialysis or ultracentrifugation.

**Table 3:** Summary of micromixers

Subtypes	Staggered herringbone mixer (SHM):^45,103,107–116^
	Twisted channel (iLiNP):^119,120^
	Dean flow:^101,122,124–129^
Products	Empty SUVs/LUVs:^101,108,109,122,126^
	Drug loaded SUVs/LUVs:^103,107,110–112,124^
	LNP:^45,114,115,119,125,129^
	Lipoplex:^113,116^
	Lipid/polymer hybrid nanoparticles:^127,128^
Cargoes	Doxorubicin:^103,107,110^
	CPD100:^111^
	Quantum dot:^128^
	Protein:^120^
	Metformin and glipizide:^112^
	siRNA/mRNA:^45,114,115,119,129^
	pDNA/ssDNA:^113,116,125,127,129^
	CRISPR/Cas RNPs system:^124^
Device	PDMS/glass:^45,101,103,107–109,115,119,120,122,126–128^
	Nanoassemblr™:^111–113,116,124,129^
	NxGen:^124,125,127,129^
Lipid compositions	PC lipid:^103,108,109^
	PC lipid & cholesterol:^101,103,111,112,122,124,126^
	PC lipid & cholesterol & PEG lipid:^107,110^
	PC lipid & cationic lipid/ionizable lipid & cholesterol & PEG lipid:^45,114,115,119,120,124,125,129^
	DOPE & DOTAP:^113^
	DOPE & DOTAP & cholesterol:^116^
	DOPE & ionizable lipid & cholesterol & PEG lipid:^114^
	Lecithin & PEG lipid & PLGA:^128^
	Cationic lipid & PEG lipid & PLGA:^127^
Alcohol phase	IPA:^122^
	Methanol:^112^
	Acetonitrile + THF:^128^
	Acetonitrile + methanol:^127^
	Ethanol:^45,101,103,107,109–111,113–116,119,120,124–126,129^

In addition to SHM, CAE and TrM, numerous alternative micromixer designs may be used for liposomal production, such as a helical microchannel or 3D-twisted geometry. For instance, Firmino *et al.*^131^ integrated MHF and 3D-twisted crossing-sectional microchannel, and they achieved 100 nm liposomes at an FRR = 1. This 50% v/v ethanol led to high lipid concentration and high mass productivity (2.27 g lipid per h). Micromixers can also couple with each other. Bokare *et al.*^132^ optimized the multi-inlet vortex mixer by printing SHM patterns in the flow channels and achieved lipid polymer hybrid nanoparticles with a diameter of 74.5 nm and ∼0.1 PDI. Shi *et al.*^123^ added lateral structures to refine micromixers by generating secondary Dean flows (Fig. 6d). They found that by adding lateral structures, the mixing processes in both T-shape and zig-zag serpentine mixers were remarkably improved, compared to the mixing in the original geometries. By contrast, little promotion was achieved on the SAR micromixer by adding lateral structures originally based on Dean flows.

### 2.4 On-chip hydration

Hydration is probably the most classic method to produce lipid vesicles.^5^ A solid surface is first coated by a lipid film by evaporating an organic solvent such as chloroform, in which the lipids are previously dissolved. This film-coated surface is flushed by the aqueous buffer solution and the shear stress leads the lipid layers to peel off, breaking and self-assembling into polydisperse and multilamellar vesicles.^18^ To achieve small unilamellar vesicles with high encapsulation efficiency and low PDI, additional processes such as freeze–thaw^133^ and extrusion^26^ are necessary. Microfluidics has been used to refine this conventional technique as the flow conditions of hydration and properties of vesicles can be more controllable.

**Fig. 7:**
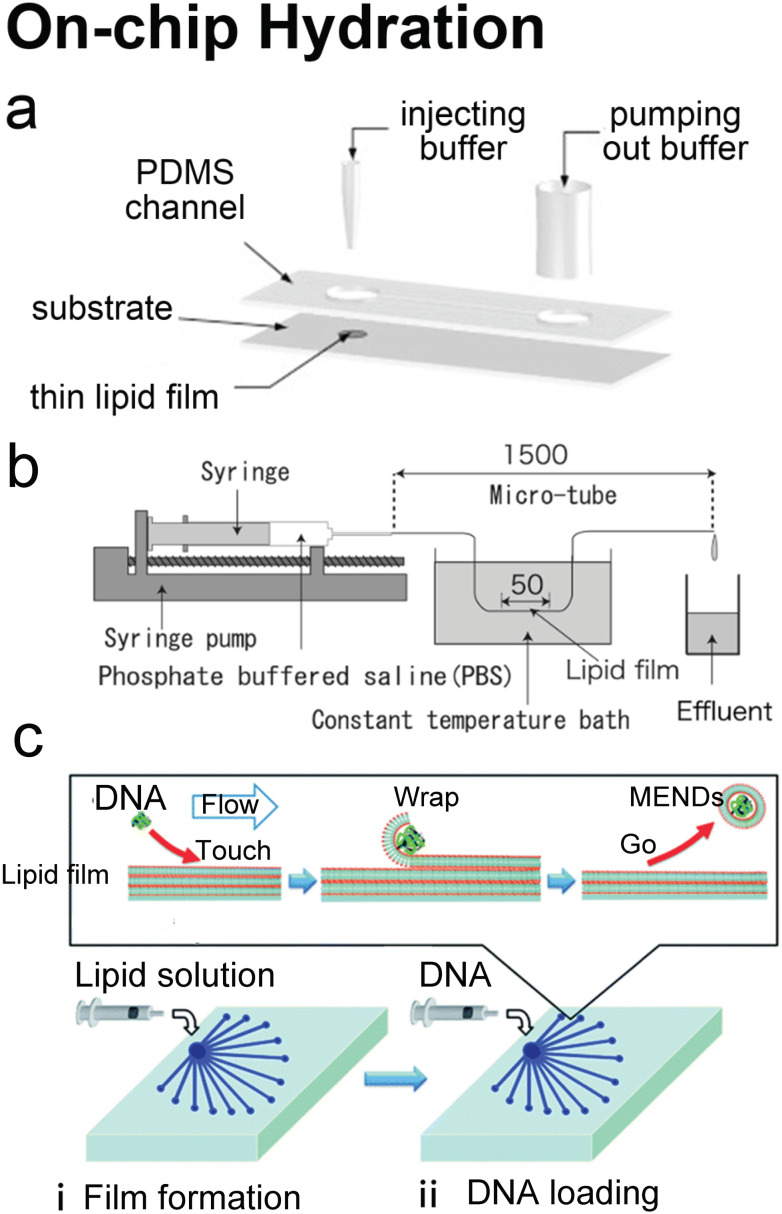
Microfluidic refinements for hydration. a| Schematic representations of the design of Y. Lin *et al.* Two 4 mm diameter wells were formed by bonding 2 mm thick PDMS to glass. The two cavities were connected by a channel. One cavity was for lipid film accommodation and hydration buffer injection to produce liposomes, and the other was for pumping out buffer. Reproduced from ref. 134 with permission from Elsevier, copyright [2006]. b| Schematic drawing of the micro-tube system designed by H. Suzuki *et al.* Lipid chloroform solution was first injected to the 50 mm position of the microtubes with the same total length of 1.5 mm and various diameters of 200, 320 and 530 μm. After the lipid film formed by desiccator drying, PBS was pumped in and washed the microtubes, and the effluent was collected. Reproduced from ref. 135 with permission from the Society of Chemical Engineers, Japan, copyright [2008]. c| Schematic illustration of K. Kitazoe *et al.*'s touch-and-go lipid wrapping technique. This technique constructed multifunctional envelope-type gene delivery nanodevices (MENDs) in two steps: (i) lipid coating in the microfluidic device and (ii) MEND formation in the microfluidic device. The top panel illustrates the mechanism of MEND formation based on the electrostatic interaction: the positively charged condensed plasmid DNA touched the lipid films on the glass, the substrate was wrapped in the lipid bilayer, and released as the MENDs. Reproduced from ref. 136 with permission from the Royal Society of Chemistry, copyright [2011].

Lin *et al.*^134^ developed a microfluidic hydration method by covering a DMPC lipid film-coated glass slide with a PDMS slide, which had a long and narrow microchannel on it (Fig. 7a). An aqueous solution was injected to flush the lipid film in the microchannel. Lipid aggregates of different shapes and sizes including lipid vesicles, microtubes and vesicle-tubes networks could be formed by adjusting the flow rate of the aqueous stream. Similarly, Suzuki *et al.*^135^ filmed the lipids on the inner wall of microtubes (Fig. 7b). The tubes were washed with phosphate-buffered saline (PBS) for hydration. They prepared MLVs with narrower size ranges (510 nm ± 80 nm) and liposome production yield up to 39.2%. They also demonstrated that the peak sizes of their vesicles were determined by the Reynolds number so the size peak could be adjusted by the tube diameter and the bulk velocity. Kitazoe *et al.*^136^ developed a microfluidic hydration method for gene delivery applications (Fig. 7c). An aqueous buffer containing the condensed plasmid DNA cores was injected from one central inlet to hydrate the lipids film coated on multiple outlet channels in peripheral distribution. Their products, multifunctional envelope-type gene delivery nanodevices (MENDs) presented a homogeneous diameter distribution (around 200 nm). The whole procedure took less than 5 min. However, the effects of microfluidic refinement on the gene delivery function of MENDS were not reported.

Microfluidic devices can strengthen the hydration method in tuning products' size^135^ and enhancing production rate.^134^ And different from MHF and micromixers which involve using alcohol in preparing vesicles, organic solvents have been removed before hydration. Thus, on-chip hydration is ideal for preparing ‘clean’ vesicles for clinical use. However, hydration requires pre-formed lipid films, which is usually batch achieved, and the vesicles prepared by hydration usually have polydisperse lamellarity.^134,135^ For further refinement of the conventional hydration method, future microfluidic integration may focus on generating lipid films on chips and producing small unilamellar vesicles continuously. More work still needs to be done on microfluidic refinements to compete with the extrusion method, which is considered the gold standard for small vesicle preparation.

## 3 Microfluidics for the production of cell-sized lipid vesicles

### 3.1 Cell-sized lipid vesicles

**Fig. 8:**
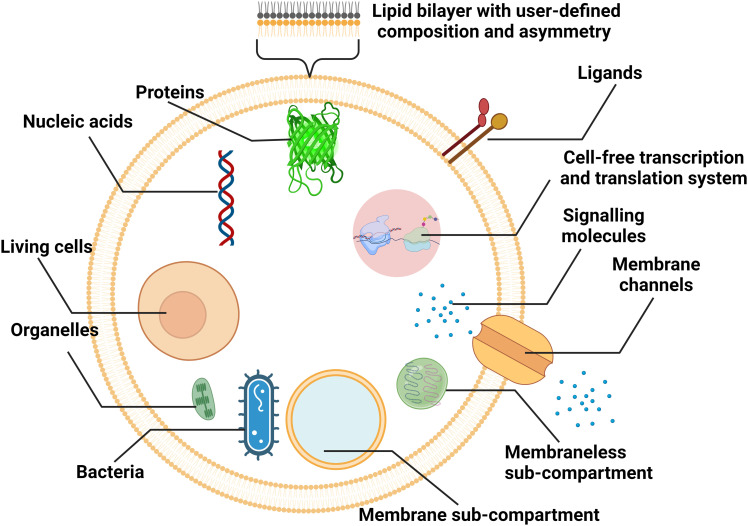
Schematic representation of cell-sized lipid vesicles. To simulate cells or function as bioreactors, ideal platforms require good encapsulation of biochemical materials, higher-order compartmentalisation, extracellular and intracellular communication, and replication of cellular metabolism.

Liposomal nanocarriers are often designed to replicate the transport mechanisms of intracellular and extracellular vesicles. With larger sizes (microscale), cell-sized liposomes, also called giant vesicles, are ideal platforms to study other aspects of cellular physiology. Compartmentalized by a lipid bilayer and incorporating biochemical motifs, cell-sized liposomes can function as microreactors hosting a diverse repertoire of biochemical reactions for synthetic biology studies, and can form the basis of artificial cells mimicking the structures, functions and behaviours of living systems from a bottom-up approach (Fig. 8).^38,137^

For cell-sized liposomes, properties like diameter, lamellarity and production rate are still significant factors for assessing preparation methods. Besides, as cell-like liposomes are often designed for tasks more complex than simple encapsulation, diverse functional features, including compartmentalisation, molecular communication and replication of cellular metabolism must be taken into consideration when producing these liposomes. In cells, spatially distinct microenvironments include numerous organelles encapsulated by a membrane. The compartment boundaries separate the interior and exterior components, across which the exchange of biochemicals allows for cellular communication and metabolism. For better simulating complex cellular functions, vesicles containing membrane and membraneless compartments have been engineered.^38,138^ Similar to biological cells, the communication in and between artificial cells relies on the transportation of signalling molecules, mainly by diffusion across lipid bilayers^139^ or through reconstituted channel proteins,^37^ and vesicle fusion.^140^ Asymmetry (where two leaflets of a bilayer membrane have different compositions) is one of the fundamental traits of biological membranes and a significant feature to pursue when engineering artificial cells, as it affects signal transduction, exocytosis, and apoptosis.^141^ In the aspect of molecular communication, some designed artificial cells are able to synthesize the signalling molecules in response to the signals they have received. The generation of signalling molecules can be conducted by constructing artificial reaction chains^142^ or by encapsulating cell-extracted or cell-free synthetic systems capable of nucleic acid and protein biosynthesis.^143,144^ More complex metabolism processes, such as continuous growth and division cycles, are attractive but still challenging for artificial cells.

### 3.2 Emulsion-based microfluidics

Many conventional methods prepare cell-sized vesicles from water-in-oil (W/O) emulsions.^29–34^ W/O emulsions are formed by the emulsification of two immiscible phases in the presence of a lipid/surfactant, where one aqueous phase of lower volume forms lipid-stabilised droplets within a bulk oil phase of a larger volume. The emulsion droplets essentially act as a template around which a membrane is assembled. In this section, we will introduce how microfluidics has been applied to improve and revolve the emulsion-based vesicle preparation by continuously generating lipid-coated emulsion templates with uniform size and forming resultant cell-sized vesicles with good encapsulation efficiency and user-defined membrane properties.

#### 3.2.1 Microfluidic refined emulsion phase transfer

**Fig. 9:**
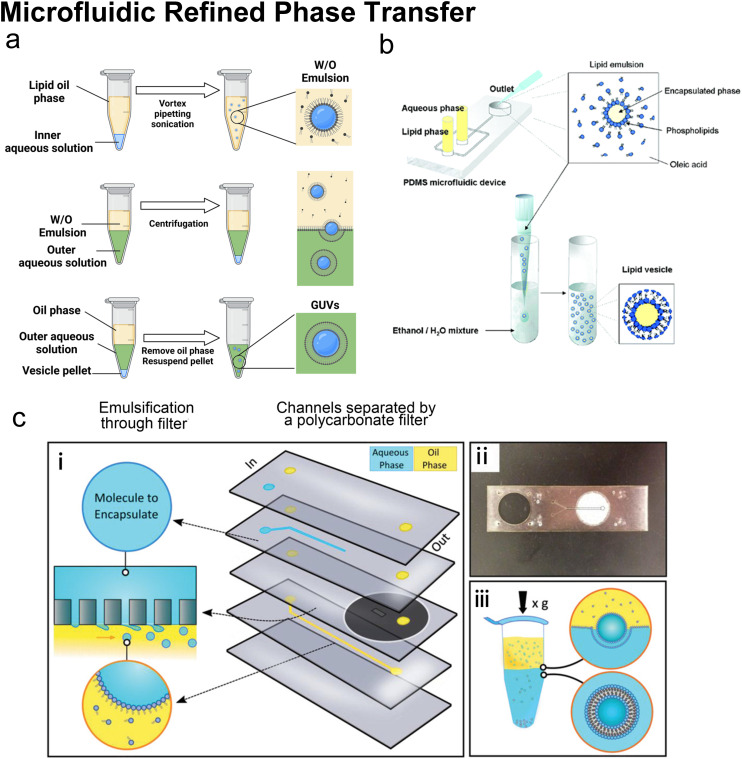
Microfluidic refined phase transfer. a| Mechanism of bulk emulsion phase transfer. W/O emulsion is first generated by mixing the lipid oil phase and the inner aqueous solution (usually sucrose buffer). Then the emulsion is transferred onto the top of the outer aqueous solution (usually glucose buffer). After centrifugation, the oil phase is removed, and the pellet is resuspended to yield GUVs. b| Schematic of vesicle preparation through microfluidic emulsification and bulk template transfer. The aqueous phase containing the target encapsulated species is first emulsified in lipid-dissolved oleic acid for stable lipid emulsions and then injected into an aqueous mixture consisting of ethanol and water to remove the oleic acid. Reproduced from ref. 145 with permission from the American Chemical Society, copyright [2006]. c| A microfluidic device for generating GUVs or LUVs in two steps. (i) Schematic of the different layers used to create the final microfluidic device. An aqueous solution containing molecules to encapsulate is pumped into the first input channel (blue). Oil solvents saturated with lipids are pumped into the second input channel (yellow). These two channels are separated by a layer of polycarbonate filter. Droplets are formed by driving the aqueous solution through the rigid filter into the oil phase under cross-flow emulsification conditions. (ii) An image of a single microfluidic device. The outlet channel has been outlined to help with visualization. (iii) Emulsion phase transfer of lipid-stabilized microscale or nanoscale droplets through a lipid-rich interface to form GUVs or LUVs. Reproduced from ref. 146 with permission from John Wiley and Sons, copyright [2019].

When preparing GUVs by conventional emulsion phase transfer (EPT) (Fig. 9a), the W/O emulsions are originally generated by mixing an aqueous phase and a lipid-containing organic phase utilizing bulk mixing processes such as vortex,^31,32^ pipetting^33^ or sonication.^34^ These lipid monolayer-coated W/O droplets are passed through a second oil–water interface, which is stabilized by phospholipids, to generate the outer leaflet lipid layer. Making use of a density difference between the aqueous droplets and oil medium, centrifugation is widely applied to complete the lipid bilayer and remove the oil.^32–34^ The vesicles are finally collected from the bottom aqueous phase. As the vesicles' sizes strongly depend on the sizes of the initial W/O droplets, the size distribution of the resultant GUVs is usually poor due to uncontrolled template generation steps such as vortex and pipetting. Besides, the oil left in the samples prepared by conventional EPT is usually non-negligible and may lead to aggregations and GUV defects.

Microfluidics was initially combined with EPT to address its problem of polydisperse sizes^145,147,148^ as the droplet generation on microfluidic chips has uniform size distribution and high production rates. Tan *et al.*^145^ (Fig. 9b) and Nishimura *et al.*^147^ generated W/O droplets in typical flow-focusing geometries where two immiscible phases were injected orthogonally. The aqueous phase dispersed into droplets and the organic phase played the role of a droplet carrier. Tan *et al.*^145^ stabilised their lipid-coated droplet templates with oleic acid, which was removed by injection into a mixture of ethanol and water. They encapsulated various biological species in the vesicles, ranging from HeLa cell-cervical carcinoma cells, micron-sized fluorescent beads, to nanosized GFP. The mean diameters of these three kinds of vesicles were 62.4 μm (∼20% variation), 55.9 μm (∼10% variation) and 27.2 μm (∼20% variation). Nishimura *et al.*^147^ investigated the effect of droplet templates and centrifugal process on the size distribution of resultant GUVs. With optimal template sizes and centrifugal conditions, GUVS with a desired size (tunable diameter between 6.5 and 13.5 μm) and a narrow size distribution (low to 32% variation, 43% for vortex method) were obtained. They also found that supplementation of nonionic detergents could improve the size control on both the droplet templates and the GUVs. Romanov *et al.*^146^ used a polycarbonate filter to separate the channels of oil and water, which allowed the simultaneous formation of multiple W/O droplets (Fig. 9c). The size of the W/O templates depended on the filter pore size and the wall shear stress, which led to tuneable template-dependent diameters of the resultant vesicles, ranging from ∼10 μm to ∼100 nm. The resultant vesicles supported the assembly of asymmetric bilayer leaflets and transmembrane protein (alpha-hemolysin) insertion. The degree of asymmetry was found to be affected by oil properties. These three studies all used centrifugation to transfer W/O droplets into vesicles. Good encapsulation efficiency^145,146^ and size control^145–147^ were reported.

Kuroiwa *et al.*^149,150^ developed another partly microfluidic emulsion-based method, namely the ice droplet hydration method. As this name indicates, the droplets generated by microfluidics were frozen first, and then these ice droplets were extracted from the organic phase by sedimentation. The organic phase was separated as supernatant and removed by rotary evaporation. After hydration recovery in an aqueous medium, the lipid-stabilized ice droplets were transferred to giant vesicles. Ice droplets could avoid extensive water droplet coalescence and lead to monodisperse vesicle sizes tuned by their starting water droplets. However, the application of ice droplet transfer was limited by its low encapsulation efficiency (35%) and uncontrollable lamellarity (mainly multilamellar). If these giant vesicles prepared by ice droplet hydration were extruded to produce LUVs, the encapsulation efficiency would decline from 35% to 12%.^149^

#### 3.2.2 Microfluidic single emulsion transfer

The protocols described in the last section are partly microfluidic because these microfluidic-generated droplets still need bulk processes like centrifugation to form vesicles. Numerous explorations have been conducted to make the droplet emulsion transfer methods completely microfluidic. One approach is to apply microfluidic control to the “enveloping” process which converts the W/O droplets into vesicles. As this approach only has W/O emulsions as intermediates, this approach is defined as ‘microfluidic single emulsion transfer’.

**Fig. 10:**
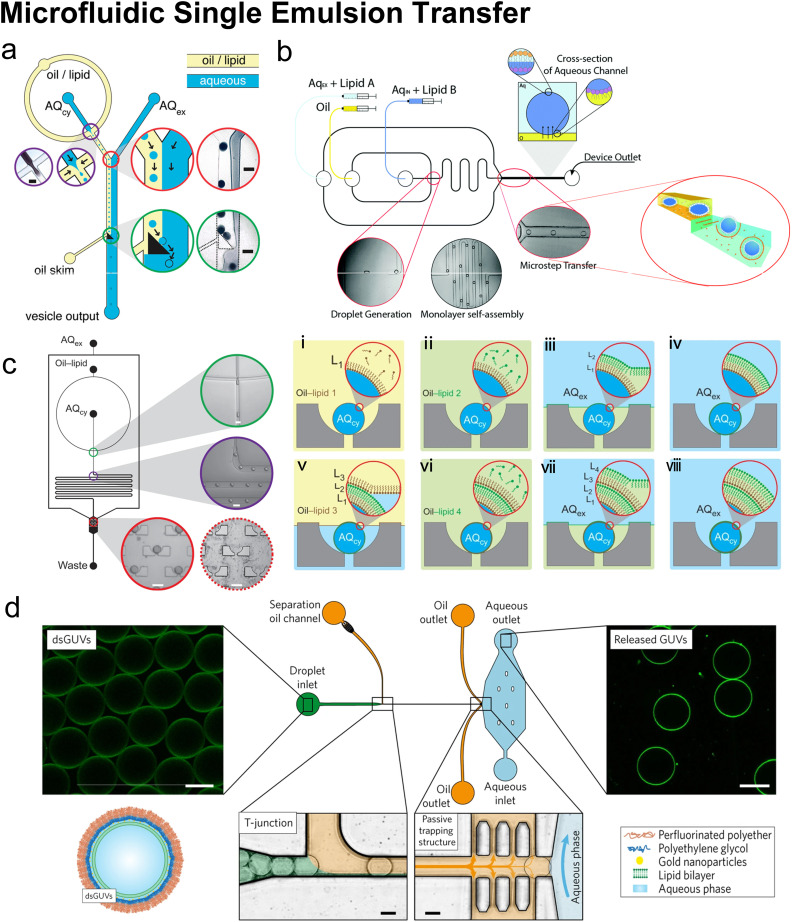
Microfluidic single emulsion transfer. a| Schematic of microfluidic droplet transfer assisted by a triangle post. The triangular post skimmed the oil flow and deflected the preformed W/O droplets along its hypotenuse into the extracellular aqueous phase (AQ_ex_). As droplets traverse the interface, a second lipid monolayer is coated and GUVs are formed (micrograph scale bar = 100 μm). Reproduced from ref. 151 with permission from the American Chemical Society, copyright [2011]. b| 2D schematic of microfluidic droplet transfer assisted by micro-step. The W/O droplets were transformed from the oil channel into a wider and deeper aqueous channel, where they picked up a second lipid monolayer from small vesicles in the Aq_ex_. Reproduced from ref. 153 and 154 with permission from the Royal Society of Chemistry, copyright [2015 and 2016]. c| Schematic of microfluidic droplet transfer assisted by hydrodynamic traps (left). W/O droplets were generated by focusing flow, travelled through the delay line, and trapped by an array of cups. Schematic of layer-by-layer assembly (right (i)–(viii)) new phase boundaries were successively driven over the trapped droplets, and new lipid monolayers were deposited (micrograph scale bar = 100 μm). Reproduced from ref. 152 with permission from Springer Nature, copyright [2013]. d| Formation and analysis of droplet-stabilized GUVs. The copolymer-stabilized W/O droplets (dsGUVs) were separated at a T junction by a tributary oil flow containing 20 vol% destabilizing surfactants. The passive trapping structures drained the oil phase into adjacent outlets, and GUVs were released as the droplets entered the aqueous phase. Scale bars, 20 μm. Reproduced from ref. 155 with permission from Springer Nature, copyright [2017].

Hydrodynamic trapping is one of the most efficient strategies to transfer W/O emulsions into vesicles on microfluidic chips.^151–155^ For example, Matosevic *et al.*^151^ set a triangle post near the co-flow junction to skim the oil phase and led the deflected droplets to pass through the lipid-stabilized W/O interface (Fig. 10a). 83% encapsulation efficiency was obtained when loading small-molecule fluorescein (FAM, 332 Da). In a later publication by the same group,^152^ a layer-by-layer (LbL) assembly protocol was reported, in which droplets were fixed by hydrodynamic trap arrays and a second lipid monolayer was deposited on these droplets actively (Fig. 10c). Through LBL assembly, the encapsulation efficiencies of small-molecule fluorescein and macromolecular dextran (10 kDa) were enhanced to over 90%. The microfluidic LbL strategy also presented potential for fabricating asymmetric membranes and multilamellar vesicles, as it later presented in a bulk EPT analogue.^156^ Elegantly, fluorescent quenching of NBD labelled on the tails or heads of lipids was used to probe the lamellarity of intermediates and final products. Karamdad *et al.*^153,154^ set a ‘step junction’ to transfer the lipid-coated single emulsion into GUVs with a lipid bilayer (Fig. 10b). At the step junction, the channel geometry became deeper from 50 μm to 100 μm, which made the emulsions fall into the deeper hydrophilic channel. The aqueous solution in the deeper hydrophilic channel contained small vesicles and served as the lipid source of the outer monolayer of GUV products.^153,154^ Weiss *et al.*^155^ introduced a tributary oil flow at a T junction to separate the droplets and constructed rows of pillars to guide and decelerate the droplet flow (Fig. 10d). The oil was drained into adjacent oil outlets. Thus, as the droplets entered the aqueous phase, they were transferred to GUVs.

The trapping strategy requires precise control of geometric design and microfluidic conditions. The shape and position of the trapping barrier must be meticulously designed to avoid droplet bursting, and to reduce the amount of residual trapped oil as much as possible.^151^ The flow rate should produce proper pressure at the oil/water junction.^155^ The throughput of vesicle production is determined by the rate of trapping droplets rather than the rate of generating droplets because the upper limit of the trapping rate is always several orders of magnitude smaller than that of the generating rate. Therefore, higher flow rates increase the rate of droplet generation but do not necessarily lead to higher vesicle production throughput. Instead, the high relative rate of droplet formation can result in high shear forces near the trapping barrier leading to the bursting of droplets.^151,152^ Beyond manipulating droplets and forming GUVs, the trapping strategy has also been widely used in immobilising single vesicles.^157,158^

As mentioned above, in addition to forming a lipid monolayer at the water/oil interface with the lipids dissolved in oil, which is defined as the ‘lipid-out’ approach, forming a monolayer through the fusion of small vesicles from the aqueous phase onto the interface is known as the ‘lipid-in’ approach.^159^ Hwang *et al.*^159^ named this ‘lipid-in’ approach in forming asymmetric droplets interface bilayer (DIB). Compared with the lipid-out approach, the lipid-in approach allows a broader range of membrane compositions because some lipid-like molecules have poor solubility in specific oils, such as lipopolysaccharides. Also, these small vesicles are ideal vehicles for membrane proteins, known as proteoliposomes, whose reconstitution onto GUVs can be completed simultaneously when the bilayers of GUVs are formed. Karamdad *et al.*^154^ used this lipid-in strategy to fabricate asymmetric vesicles. Two different lipid compositions were adsorbed onto the W/O and O/W interfaces respectively so that two monolayers containing different lipid compositions coated the droplets in succession (Fig. 10c). Weiss *et al.*^155^ developed the ‘lipid-in’ strategy into droplet-stabilized GUVs (dsGUVs) technology (Fig. 10d). To address the mechanical and chemical instability of the lipid-based compartment, lipid vesicles, either LUVs or GUVs, were encapsulated in copolymer-stabilized droplets and fused to form a supported lipid bilayer at the copolymer-stabilized droplets' inner interface. Pico-injection was used to induce Mg^2+^ to trigger the fusion and deliver biological materials like transmembrane proteins and cytoskeletal proteins. The copolymers were removed by a tributary oil flow containing 20 vol% destabilizing surfactants. With the help of passive trapping structures, the oil phase was drained, and GUVs were released with no oil or surfactants remaining.

#### 3.2.3 Microfluidic double emulsion-based vesicle generation

**Fig. 11:**
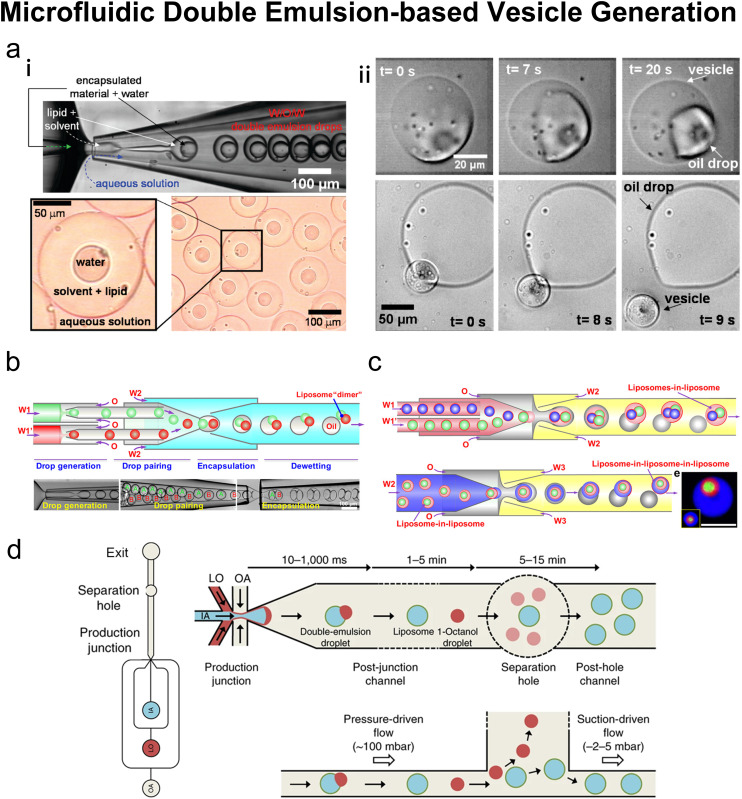
Microfluidic double emulsion-based vesicle generation. a| (i) Top: Formation of phospholipid-stabilized W/O/W double emulsion in a glass microcapillary device. Bottom: Optical micrograph of the double emulsion collected. (ii) Top: Vesicle formation through solvent drying on the vesicle surface. Excess phospholipid is concentrated in the remaining oil drop attached to the resulting vesicle. Bottom: Release of a vesicle from a double emulsion drop pinned on a glass slide. The oil drop that contains excess phospholipids remains on the glass slide. Reproduced from ref. 163 with permission from the American Chemical Society, copyright [2008]. b| Fabrication of liposomes with distinct multicompartments. Schematic (top) and snapshots (bottom) of the fabrication of double emulsions with two distinct droplets. Scale bars are 100 μm. Reproduced from ref. 167 with permission from the American Chemical Society, copyright [2016]. c| Top: Schematics of the microfluidic preparation of double emulsions with distinct interior liposomes (liposomes-in-liposome) and the dewetting process. Bottom: The formation of triple vesosomes (liposome-in-liposome-in-liposome) and the resultant structures. Scale bars, 100 μm. Reproduced from ref. 168 with permission from the American Chemical Society, copyright [2017]. d| Schematics showing octanol-assisted liposome assembly (OLA) vesicle production and purification. An overall layout of the microfluidic device and the post-junction channel (left). A top view (right top) and a side view (right bottom) of the OLA junction. IA, inner aqueous phase; LO, lipid-carrying organic phase; OA, outer aqueous phase. Reproduced from ref. 169 with permission from Springer Nature, copyright [2016].

The second approach towards completely microfluidic emulsion-based liposome preparation is directed to the double emulsion-based vesicle generation method. As its name indicates, this method uses water-in-oil-in-water (W/O/W) double emulsions as precursors for lipid vesicle generation. Before integrating with microfluidics, the generation of W/O/W double emulsions has been well studied in bulk.^160^ Typically, preparing a W/O/W droplet involves the formation of an oil-in-water emulsion outside a water-in-oil emulsion, just like encasing a bubble within another bubble. Monodisperse W/O/W emulsions^161^ and higher order emulsions^162^ had been produced by microfluidic devices before they were used as templates for vesicle generation. However, the double emulsion method had not demonstrated its potential to be a practical method for liposome preparation until Shum *et al.*^163^ reported using glycerol-assisted slow evaporation to remove the intermediate oil phase. Using a glass-capillary microfluidic device, GUVs with diameters ranging from 20 μm to 70 μm were produced at the rate of 500 Hz (Fig. 11a (i)). W/O/W emulsions were transferred into GUVs through the dewetting phenomenon, where an oil-in-water droplet was squeezed out between the W/O interface and O/W interface (Fig. 11a (ii)). The properties of the GUVs could be well controlled by their double emulsion templates. Shum and co-workers further investigated the dewetting-induced formation of spherical and multicompartmental polymersomes in the following publications.^164,165^ This squeezing process is not driven by any external mechanical forces but by the adhesion between the two interfaces. The oil composition in the solvent mixture, such as chloroform and hexane, plays a vital role in this adhesion. Upon the selective removal of the chloroform solvent used to dissolve amphiphilic diblock copolymers, the copolymer concentration increased to a critical concentration and adhesion was triggered. Based on the oil formula proposed by Shum *et al.*,^164^ Arriage *et al.*^166^ dissolved two different lipid compositions separately. The two oil solutions were injected individually to form water-in-oil-in-oil-in-water triple emulsions so that an asymmetric bilayer could be built after solvent removal. Asymmetric GUVs with asymmetry up to 70% were produced at the frequency of 200 Hz.

To improve the control over dewetting and oil removal, Deng *et al.* added surfactants (Pluronic F-68) in the outer water phase to minimize the interfacial energy.^167,168^ Based on this surfactant-assisted dewetting, liposomes consisting of a multitude of coupled compartments could be created at the rate of 1000 Hz (ref. 167) (Fig. 11b). Also, vesosomes (liposome-in-liposome structure, Fig. 11c) presented uniform size (mean diameter was 43 μm for internal liposomes and 102 μm for external liposomes) and allowed concentric, pericentric and multicompartmental structures.^168^*In vitro* transcription (IVTx) mix and *in vitro* transcription/translation (IVTT) were encapsulated in different compartments to mimic intracellular compartmentalisation, and membrane nanopores (melittin) were reconstituted for communication between these compartments. Deng *et al.* also used their microfluidic double emulsion method to encapsulate membraneless coacervate organelles in GUVs.^170^ Coacervates, also called condensates, are water droplets in water, formed by spontaneous liquid–liquid phase separation (LLPS) in an aqueous solution containing two oppositely charged polyelectrolytes.^171^ In cells, some biomolecules, such as proteins or nucleic acids, undergo LLPS to form condensates.^172^ Deng *et al.* presented the collection and release of DNA by reversible thermo-sensitive coacervation of macro-ions in liposomes and the spatial organisation of *in vitro* transcription.^170^

Besides the constrained mixture of chloroform and hexane, 1-octanol was reported to be an alternative oil for dewetting transition, based on which octanol-assisted liposome assembly (OLA) was developed^169,173,174^ (Fig. 11d). As the interfacial energy was minimized, the 1-octanol pocket in the double emulsion split off quickly and the oil was removed as the bilayer zipped up. Deshpande *et al.* pioneered the development of OLA.^169,173^ They managed to produce liposomes with a diameter as small as 5–20 μm and with a size variation as small as 3% at the rate up to 75 Hz. They also integrated the OLA platform with a subsequential physical splitter to divide the cell-like liposomes.^175^ Deformed by a Y-shaped bifurcation, remarkably, the liposomes produced by OLA were uniformly divided into two stable daughter liposomes. Tivony *et al.*^176^ integrated on-chip production and purification of OLA GUVS. Various residues were separated from the giant vesicles through stream bifurcation with an efficiency high up to 0.99. Schaich *et al.*^174^ investigated the lipid composition in the vesicles generated by OLA, which matched the input lipid composition in the octanol phase. The OLA vesicles also presented quantitatively similar lateral lipid diffusion coefficients, as compared to vesicles generated by electroformation. OLA was also used to form GUVs containing coacervates. Deshpande *et al.*^138^ achieved spatiotemporal control on coacervates formation in OLA-GUVs by triggering LLPS with passive molecular diffusion through pores or active enzymatic polymerization of nucleic acids. Last *et al.*^177^ fabricated pH-controlled coacervates in OLA-GUVs, and found that the interactions between the coacervates and lipid membrane were significantly affected by the electrostatic and hydrophobic properties of the membrane.

Oleic acid is another organic solvent named for the role of the intermediate oil phase in double emulsion.^178–180^ Similar to what Tan *et al.* proposed in their microfluidic-refined EPT method,^145^ ethanol is used to extract oleic acid from W/O/W emulsions and force the two lipid monolayers to bond together. The postprocessing of collected samples is not complex because a flat layer of extracted oil will be suspended in the solution and the ethanol could be eliminated by evaporation.^179^ Using oleic acid as the solvent, Lu *et al.* prepared asymmetric GUVs with the ‘dual pinching’ separation strategy.^179^ Two kinds of lipids were added successively through two oil channels. Triangle posts were set near the divaricating channels to split the first oil phase surrounding the emulsions into these channels as waste. So that the second lipid solution could replace the first lipid solution. However, the main problem of using oleic acid is that the extraction process may take as long as 18 hours to remove all oleic acid.^179^

#### 3.2.4 Continuous droplet interface cross encapsulation (cDICE)

**Fig. 12:**
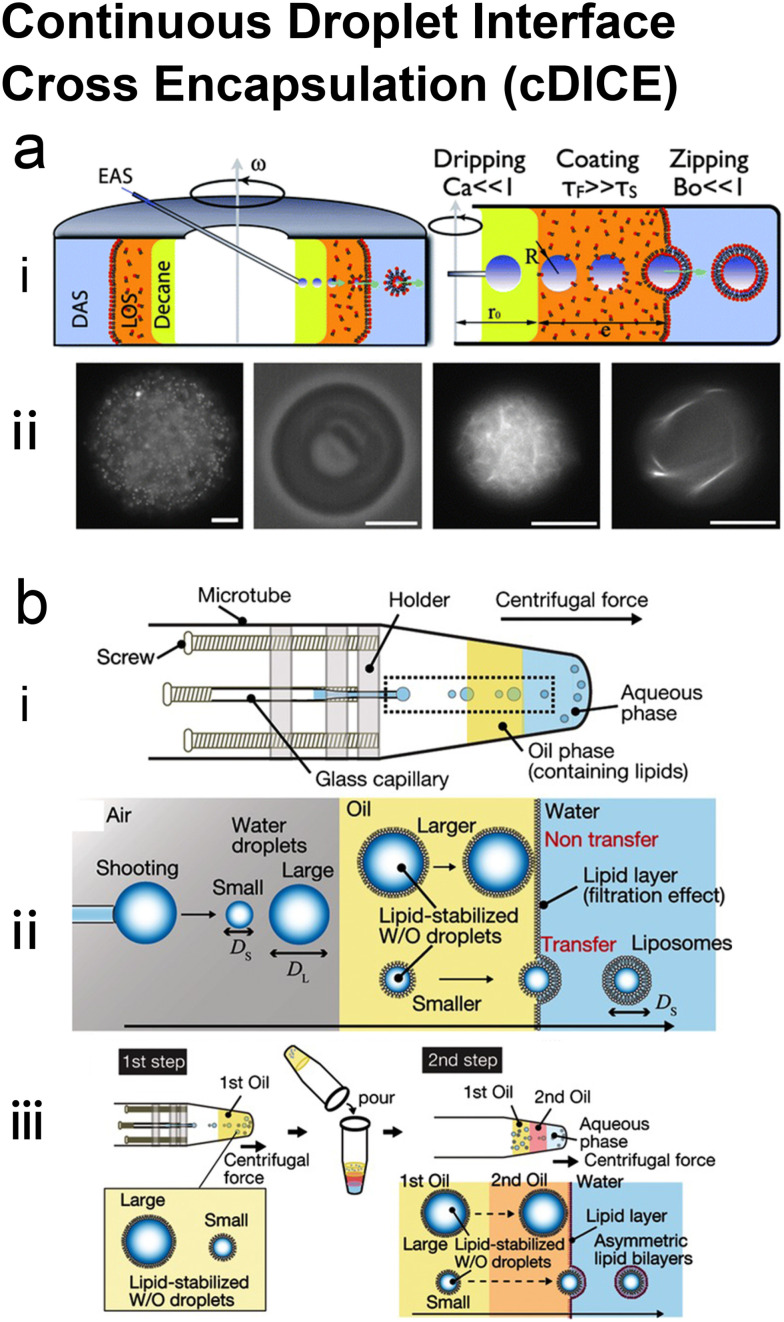
Continuous droplet interface cross encapsulation (cDICE). a| (i) Schematic side view and working conditions of the cDICE setup. Abbreviations and physical variables are explained in the body text. (ii) Examples of the suspensions encapsulated in the vesicles. From left to right: 1-micron polystyrene colloids at 4% v/v, red blood cell, thin and thick actin filament bundles with fascin. The scale bar is 10 mm in all panels. Reproduced from ref. 181 with permission from the Royal Society of Chemistry, copyright [2011]. b| Formation of GUVs by the droplet shooting and size-filtration (DSSF) method. (i) Capillary-based microfluidic device. (ii) Generation of GUVs and mechanism of size-filtration (within the rectangle shown in (i)). (iii) Two-step preparation of asymmetric GUVs in DSSF. Reproduced from ref. 184 with permission from John Wiley and Sons, copyright [2015].

Continuous droplet interface cross encapsulation (cDICE) is a variant of double-emulsion, reported by Abkarian *et al.*^181^ Different from the microfluidic double emulsion methods mentioned above which rely on dynamic flow mixing to generate lipid-stabilized emulsions and vesicle products, cDICE is more like conventional bench phase transfer, using centrifugal force to transfer capillary-generated W/O droplets across a lipid monolayer at the oil–water interface^182,183^ (Fig. 12a). Briefly, in cDICE, an empty dish was first set rotating to generate centrifugal forces and then desired volumes of the dispersing aqueous solution (DAS), the lipid-in-oil solution (LOS) and decane were added sequentially. Under centrifugation, the three solutions remained separated as outer, intermediate and inner layers due to their density differences. The encapsulated aqueous solution (EAS) was injected by capillary at a constant rate. After travelling through the three layers, the droplets formed GUVs and were collected from the outer DAS. Compared with microfluidic-refined emulsion phase transfer methods, cDICE has got rid of batch centrifugation and facilitated continuous production.

As Abkarian *et al.* stated,^181^ there were three main steps for the production of GUVs in cDICE. First, droplets were generated by dripping off the capillary, during which the size of droplets could be tuned by capillary diameter and the capillary number Ca.5
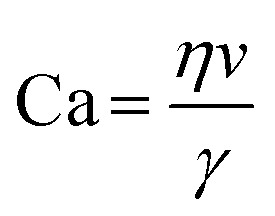
where *η* and *v* are the viscosity and velocity of the fluid and the *γ* is the interfacial tension between LOS and DAS. When Ca was set at 0.08, the droplet size was around three times the capillary size with a PDI of 11%. The inner alkane layer of a lower viscosity such as decane could also improve size distribution. Second, the time droplets travel in the LOS (*τ*_F_) must be longer than the characteristic lipid adsorption time (*τ*_S_) such that the lipid could tightly pack at the droplet surface before reaching the interface between LOS and DAS. To achieve this, the LOS layer thickness was adapted depending on the kinetics of lipid adsorption. Finally, as the droplets crossed the LOS/DAS interface, the zipping of the monolayer of the droplet and the monolayer at the interface favoured a non-inertial regime. The Bond number Bo could be used to compare inertia and interfacial tension.6
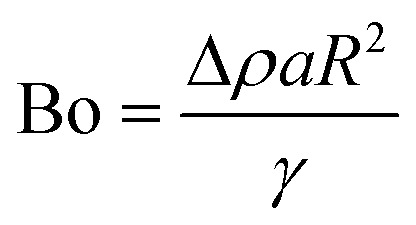
As the Bo was small in both LOS and DAS, the droplets were mainly affected by interfacial tension while the inertia would not significantly deform the monolayer. However, if the ‘healing’ of the lipid monolayer between LOS and DAS after the passage of one droplet was not completed before the next passage, the subsequent droplet would burst in DAS, which might explain why the GUV yield was only 40% in this prototype.^181^

Van de Cauter *et al.*^185^ optimised the cDICE protocol by tightly controlling the environmental conditions and tuning the lipid-out dispersion. They found that humidity control played a significant role in generating clean GUVs. Using a dehumidified environment (30%), such as in a glove box or using a dehumidifier, to prepare and store lipid-in-oil dispersion and perform the cDICE experiments, led to a robust formation of clean GUVs. They also improved the encapsulation efficiency by adjusting the organic solvents and lipid types. The decane-based dispersion presented better G-actin encapsulation than the chloroform-based dispersion as the decane facilitated faster lipid adsorption. When encapsulating a cell-free expression system (PURE), the addition of PEGylated lipids, even 0.01 mol%, greatly enhanced the expression level. To avoid clogging, the narrow glass capillaries used in the prototype^181^ were replaced by commercially available fused silica capillary tubing with larger diameters. Using silica capillaries, the GUVs were formed more reliably with high encapsulation efficiency but a relatively broader size distribution (47% variation).

Blosser *et al.* expanded the use of cDICE to more complex lipid compositions.^186^ They found that cDICE could effectively produce monodisperse GUVs containing a high percentage of charged lipids in ionic solutions. Vesicles could be prepared using a mixture of two negatively charged lipids (50 : 50 DPHPG : DPPG) at an ionic buffer condition comparable to physiological levels. Different from good incorporation of the charged lipids, Blosser *et al.* found cholesterol in the LOS layer unable to be incorporated into the vesicles substantially (<10%). Cholesterol is more hydrophobic than phospholipids as it has only one hydroxyl head group, which leads to a higher solubility in the oil phase but harder partitioning into the lipid monolayer at the interface. Blosser *et al.*'s solution was cholesterol-loaded methylated β-cyclodextrin (mβCD), a molecule which could add cholesterol into the vesicles previously formed by cDICE. To achieve the one-step incorporation of cholesterol, Dürre *et al.*^183^ added a second oil layer between the DAS and LOS, in which the amount of mineral oil is minimized by replacing mineral oil with silicone oil. The addition of silicone oil reduced the solubility of cholesterol in the oil phase and improved the cholesterol incorporation efficiency to about 25–50%. The emergence of GUV phase separation was greatly improved by this so-called ‘double-layer cDICE’ method. In an earlier report, a variant of cDICE developed by Morita *et al.*,^184^ namely droplet shooting and size-filtration (DSSF), enabled direct incorporation of cholesterol during the formation of GUVs. DSSF also relied on centrifugally propelling droplets across the oil–water interface. In DSSF, the GUVs were generated in spinning microtubes (Fig. 12b). Thus, compared with cDICE using dishes, DFSS required smaller volumes but accordingly produced fewer GUVs. Blosser *et al.*^186^ thought that the small-volume property of Morita *et al.*'s DSSF,^184^ as well as the long incubation time, was favourable for cholesterol's incorporation. As the DSSF's full name indicated, Morita *et al.* mentioned a size filtration effect during the emulsification, by which the oil–water interface could hold back big droplets and selectively let small droplets transfer through to form GUVs. Morita *et al.* also reported a two-step preparation of asymmetric GUVs in the same paper,^184^ in which emulsions were first generated by centrifugal droplet shooting and then another lipid monolayer was coated by only centrifugation.

GUVs produced by cDICE have presented great potential in encapsulating wide types of materials, including micro colloids,^181^ proteins,^181,183–185,187,188^ nucleic acids,^185^ cell-free protein expression system,^184,185^ SUVs,^185^ living cells^181^ and bacteria.^185^ It is worth mentioning that, since cDICE was invented, it has been used to investigate cytoskeletal networks in artificial cells by encapsulating actin cortex.^181,183,185,187,188^ Keber *et al.*^182^ encapsulated microtubules and molecular motors to form an active nematic film in cDICE-produced GUVs. The shape of the vesicles could be controlled by topological constraints.

Compared with other emulsion-based microfluidic methods mentioned above which were based on PDMS chips or complex capillary systems, cDICE is easier and cheaper to setup and operate. Facilities requiring heavy investment, such as clean rooms and CNC drilling machines, are not necessary for cDICE in device fabrication or experimental operation. Also, the GUVs produced by cDICE are considered to be defect-free,^185^ as they have uniform size at optical length scales and contain no visible lipid pockets. Thus, cDICE has presented the promising potential to become a standard procedure alternative to bench EPT for most chemistry or biological labs. Like conventional bench EPT, cDICE uses oil with a high viscosity, represented by mineral oil and silicone oil.^181,183,186^ However, charged lipids do not readily dissolve in mineral oil while cholesterol so preferentially stays in mineral oil. These components may not partition into monolayers at the interface as efficiently as natural phospholipids like DOPC and POPC.^183,186^ The limit of their final percentage in the GUVs may prevent the membrane from being more physiologically relevant. To introduce lipids with bad solubility in mineral oil more efficiently, the ‘lipid-in’ approach we mentioned above might be a good choice. Assuming the monolayers are formed by vesicle fusion from the aqueous phase, the GUVs should inherit the lipid composition of the vesicles.

#### 3.2.5 Summary and scope (Table 4)

The use of microfluidic devices reduces the size dispersity of droplet templates, leading to increased control of vesicle size distribution compared to bulk droplet methods.^145,147,150,163,178,179,185^ When the two lipid monolayers of the vesicle membrane are formed independently in emulsion-based vesicle preparation, asymmetric membrane structures can be efficiently fabricated.^152–154,166,167,179,184^ Since the size and production rate of droplets are adjustable, it is feasible to encase more than one small droplet in a large droplet by microfluidic emulsion-based methods. These higher-order emulsions result in higher-order membrane structures such as vesosomes,^168^ and multi-compartment vesicles.^167,189^

**Table 4:** Summary of microfluidic emulsion-based methods

Subtypes	Microfluidic refined emulsion phase transfer:^145–147,149,150^
	Microfluidic single emulsion transfer:^151–155^
	Microfluidic double emulsion-based vesicle generation:^138,163,166–170,173,174,177–180^
	Continuous droplet interface cross encapsulation:^181–188^
Products	GUVs:^145,147,151,153,155,163,169,173,174,178,180–188^
	LUVs:^146^
	Asymmetric GUVs:^146,152,154,166,179,184^
	Multilamellar giant vesicles:^149,150,152^
	Multicompartmental liposomes:^167^
	Vesosomes:^168^
	Coacervate-contained GUVs:^138,170,177^
Encapsulation	GFP:^145,183^
	Beads:^145,187^
	Cells:^145,181^
	Enzyme and substrate:^147^
	Dodecahedral nano cages:^146^
	Fluorescent dyes:^146,149–153,163,167,179^
	Actin cortex:^155,181,185,187,188^
	Cell-free protein expression system:^167,168,178,185,188^
	Bacteria:^185^
	SUVs:^185^
	Microtubules and molecular motors:^186^
	Coacervate:^138,170,177^
Reconstitution	Alpha-hemolysin:^146,152–154,167,173,179,184,185^
	Melittin:^167,168^
	F_0_F_1_-ATP synthase:^155^
Device	PDMS chips:^138,145,147,151–155,169,170,173,174,177–180^
	Polycarbonate filter:^146^
	Silicon and glass plate:^149,150^
	Capillary:^163,166–168,181–188^
	Centrifugal setup:^145–147,181–188^
Lipid compositions	PC lipids:^151–154,167,168,170,177,178,181,184,185^
	PC lipids & PE lipids:^138,145,179^
	PC lipids & charged lipids:^163,186^
	PC lipids & cholesterol:^183,186^
	PC lipids & PEG lipids:^182,185,187,188^
	PC lipids & charged lipids & cholesterol:^146,147^
	PC lipids & cholesterol & stearylamine:^149,150^
	PC lipids & charged lipids & PE lipids:^155^
	PC lipids & cholesterol & PEG lipids:^181^
	PC lipids & cholesterol & POPS & PEG lipids:^181^
	DOPC, DOPE-biotinyl:^166^
	DOPC, DGS-NTA(Ni):^185,188^
	DOPC, DOPG/DOPE/cholesterol/Lyso PC/DSG-NTA-Ni:^169,173,174^
Oil phase	Liquid paraffin containing detergents:^147^
	Mineral oil:^146,181,182,184,186^
	Mineral oil and silicone oil:^183,185,187,188^
	*n*-Hexane:^149,150^
	Dodecane or hexadecane:^151^
	Squalene:^152–154^
	FC40 oil:^155^
	Toluene and chloroform:^163^
	Chloroform and hexane:^166–168^
	1-Octanol:^169,173,174^
	Oleic acid:^145,178–180^

The theoretical encapsulation efficiency of emulsion-based vesicle preparation is 100% (ref. 147) because each drop of encapsulated aqueous solution is wrapped by the oil phase immediately during emulsification and has no contact with the outer aqueous environment. High encapsulation efficiency and minimal leakage have also often been mentioned.^145,146,151,152,163,164,181^ However, loss of encapsulated material may still occur due to bursting of droplets^151,152^ or phase changes during droplet to vesicle conversion.^149,150^ Depending on the hydrophobicity, the encapsulant may partition into the solvent phase during GUV production as well. When encasing bead-like or cellular cargoes, some droplets and final vesicles may be empty.^145^ Compared with conventional liposome preparation methods, many emulsion-based microfluidic methods are qualified as continuous production.^153,155,166,169,178,185^

The main concern of microfluidic emulsion-based methods is the purity of the system, especially regarding residual organic solvent.^148,169^ An oil phase is indispensable when forming emulsions and ideally is removed when vesicles are formed. However, with present removal strategies, it is difficult to ensure that no trace organic solvent resides between the two lipid monolayers after vesicle formation. These residual organic solvents may have a negative impact on loading drug molecules or hosting membrane proteins.^148^ Indeed, it could be due to the presence of traces of solvent that asymmetric structures with unfavourable spontaneous curvature can be generated with emulsion templates.^148^ Additionally, some surfactants used to stabilize droplets^149,167^ or modify microchannels,^169^ may increase the complexity of purifying vesicles too. Some encouraging results have been reported with channel reconstitution of alpha-hemolysin^146,152–154,167,173,179,184,185^ and melittin,^167,168^ whose function was not affected by the residual oil in emulsion-involved vesicle preparation, but further work is necessary to confirm the universality of emulsion-based liposome production in hosting membrane proteins and signalling complexes found in biological membranes. Generally, the properties of emulsion-templated vesicles, either generated by microfluidics or bulk methods, remain fairly underexplored.^148^

### 3.3 Pulsed jetting

**Fig. 13:**
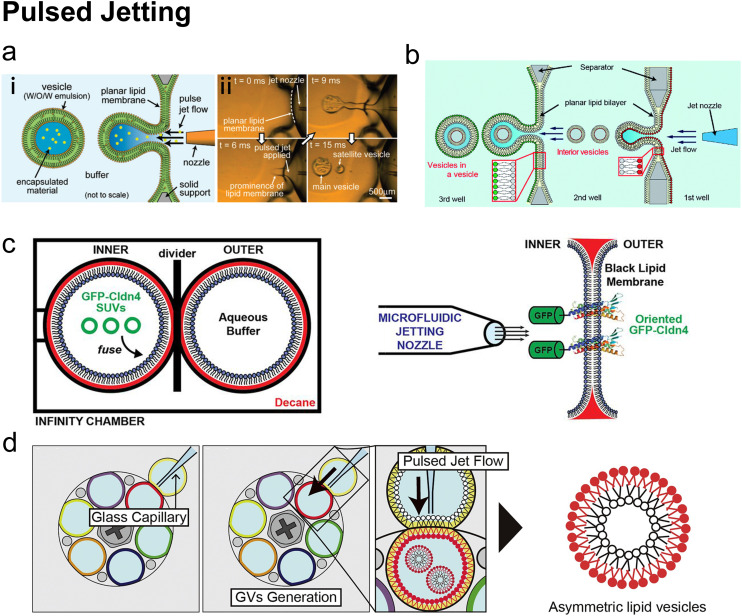
Pulsed jetting. a| (i) Conceptual diagram of the pulsed jetting method. The green area represents organic solvent. (ii) Sequential images of vesicle formation captured by a high-speed CCD camera. Reproduced from ref. 46 with permission from the American Chemical Society, copyright [2007]. b| Illustration of a mimic exocytosis system of cell-sized lipid vesicle containing small vesicles using a triple-well device. Reproduced from ref. 192 with permission from the Royal Society of Chemistry, copyright [2019]. c| Left: Two-droplet chamber configuration. SUVs delivered GFP-Cldn4 onto the lipid membrane by fusion. Right: Pulsed jetting based on lipid membrane with GFP-Cldn4 on it. Reproduced from ref. 193 with permission from Biologists, copyright [2019]. d| Schematic images of sequential asymmetric GV generation with various lipid combinations. Various asymmetric GVs could be fabricated by aligning the single outer well to inter wells containing different lipid compositions and the conducting pulsed jetting. Reproduced from ref. 194 with permission from Elsevier, copyright [2018].

Pulsed jetting is a novel microfluidic method reported by Funakoshi *et al.*^46^ When performing the pulsed jetting method, the vesicles bud out from the preformed lipid bilayer, just like the process of blowing soap bubbles^46,190,191^ (Fig. 13a). A planar lipid membrane (∼1 mm^2^) is first formed in a double-well microfluidic device, by contacting two lipid monolayers from two W/O droplets. The aqueous solution containing material for encapsulation is ejected by a pulse valve through a glass capillary nozzle against the bilayer and travels from one droplet (containing the same aqueous solution as to be ejected) to another. The jet flow deforms the planar membrane, leading to a protruded lipid tube. Contractive force due to membrane tension and the extensive inertia of the jet flow detaches vesicles from the membrane in a short timespan (10 ms).^46^ After the vesicle budding, the membrane recovers its planar state. Some satellite vesicles with smaller sizes than the main vesicles may be produced as well,^46,191^ whose formation is determined by the breakup dynamics of the resultant fluid thread. The size of the main vesicles is controlled by the dispensing time and pressure at the valve port. With the pressure fixed, the size has a positive correlation with the dispensing time.^46^ If the pressure is too small or too big, the deformation of the planar membrane will yield lipid tubes or W/O/W emulsions, respectively. Suction by glass capillary could be used to collect the GUVs from the droplet wells. The bilayer membrane of vesicles could be verified by labelling with BODIPY lipid probes, as opposed to the W/O/W emulsions which could be generated under a significantly higher actuator expansion rate.^190^

The group of Kamiya further developed the pulsed jetting method.^191,192,195,196^ They put a separator between the two planar membranes in a triple-well microfluidic device^192^ (Fig. 13b). The smaller vesicles formed by the first membrane deformation were encapsulated by the larger vesicles formed by the second membrane detachment, resulting in the formation of vesicle–invesicles. They also reported the usefulness of the pulsed jetting method in researching membrane asymmetry. Asymmetric GUVs were fabricated by adding different lipid compositions to the two wells when forming a lipid bilayer.^191,195^ Spontaneous lipid flip-flop motions were observed in the membrane of the asymmetric GUVs, which had a pure DOPC leaflet and the other leaflet comprising DOPS : DOPC at a 1 : 1 molar ratio.^191^ When flippase was reconstituted to these asymmetric GUVs by vesicle fusion from the extracellular buffer, the translocation of the PS from the outer leaflet to the inter leaflet was catalyzed.^195^ Cinnamycin, a 19 amino acid tetracyclic lantibiotic peptide which specially binds to phosphatidylethanolamine (PE) lipids can promote the flop of PE lipids, was found to promote the flop of DOPS as well from the cytoplasmic leaflet to the extracellular leaflet in the asymmetric GUVs generated by pulsed jetting. Note that this promotion of PS flop could only occur when the cytoplasmic leaflet contained both PE and PS lipids.^191^ Alpha-hemolysin (α-HL) pores were reconstituted into the outermost membrane by incubation.^191^ The successful transportation of fluorescent dyes from the outside environment into vesicles through α-HL further confirmed the unilamellarity of the GUVs. Different from reconstituting membrane proteins after pulsed jetting,^191,195^ Richmond *et al.*^196^ and Belardi *et al.*^193^ fused membrane protein-reconstituted small vesicles onto one side of the planar membrane before pulsed jetting (Fig. 13c). The bioactivity of the reconstituted proteins was also well-preserved. Recently, Gotanda and Kamiya *et al.* have reported using rotational wells to build microfluidic platforms.^194,197^ Diverse combinations of lipid compositions for the outer leaflet and the inner leaflet could be obtained on a single microfluidic chip (Fig. 13d). Armstrong *et al.* proposed high-intensity focused ultrasound from a compact acoustic lens to deform the planar bilayer, which avoided the use of nozzle.^198^

For pulsed jetting,^46^ fluorescent dyes, biomolecules and even vesicles can all be encapsulated without exposure to the outermost aqueous environment. It means the encapsulation efficiency should be high and the risk of cross-contamination would be low. Theoretically, direct encapsulation also makes pulsed jetting an ideal tool for artificial cells since organelles and biomolecules, either natural or synthetic, can be directly encased in one GUV altogether. Before that, however, efforts need to be made to ensure all encapsulated materials can withstand the high shear stress in jetting. Another advantage of pulsed jetting is in the generation of membrane asymmetry because the two monolayers of the membrane are from two different droplets. In addition to preparing cell-sized vesicles, recently, Kamiya *et al.*^199^ extended the utility of pulsed jetting towards generating nano-sized vesicles. By applying pulsed-jet flow of longer duration and higher pressure than those used for generating micro-sized vesicles, they produced vesicles of diameter ranging from 100 nm to 200 nm and membrane thickness of 5–6 nm. When preparing SUVs, compared with the conventional hydration method or MHF in which the encapsulated aqueous solution is the same as the outermost aqueous buffer, pulsed jetting has two separated aqueous compartments thus needs fewer postprocessing steps, such as centrifugation, dialysis and digestion, to remove the unencapsulated molecules or change the outermost aqueous buffer.^46^ However, the risk of contamination of the external buffer still cannot be ignored. Being able to produce both micro-sized and nano-sized vesicles is a rare feature for highly specialized microfluidic methods.

Kamiya *et al.*^191^ also evaluated the amount of residual oil in the vesicle membrane by a confocal Raman scattering microscope. They found that the molar ratio between the oil (*n*-decane) and lipid (DOPC) in the vesicles generated by their pulsed jetting method was below 0.5 (molar ratio) but had little effect on the stability and membrane dynamics. Enhancing the pressure and application time may reduce the residual organic solvents^200^ but may cause other negative effects, such as potential damage to the materials to be encapsulated. Compared with other microfluidic methods, pulsed jetting has exhibited disadvantages as it is less continuous, reproducible, and rapid. The planar membrane in pulsed jetting is often formed by batch manipulation, and continuous collecting GUVs or supplying new lipids has not been reported. The reproducibility of pulsed jetting is limited, mainly due to the irreproducible positioning of the nozzle after each reformation of the planar membrane.^194,197^ Compared with the cDICE method mentioned above, the equipment of pulsed jetting is more specialised.^186^ However, the bilayer renewal time after each jetting is longer than the monolayer renewal time in cDICE180 or the on-chip generation of W/O/W emulsion in the microfluidic double emulsion method. Thus, the production rate of pulsed jetting is lower (∼4 Hz).^46^ Integrating bilayer generation and vesicle collection into one microfluidic device could make the jetting approach more automatic and reach continuous rapid production.

### 3.4 On-chip electroformation

**Fig. 14:**
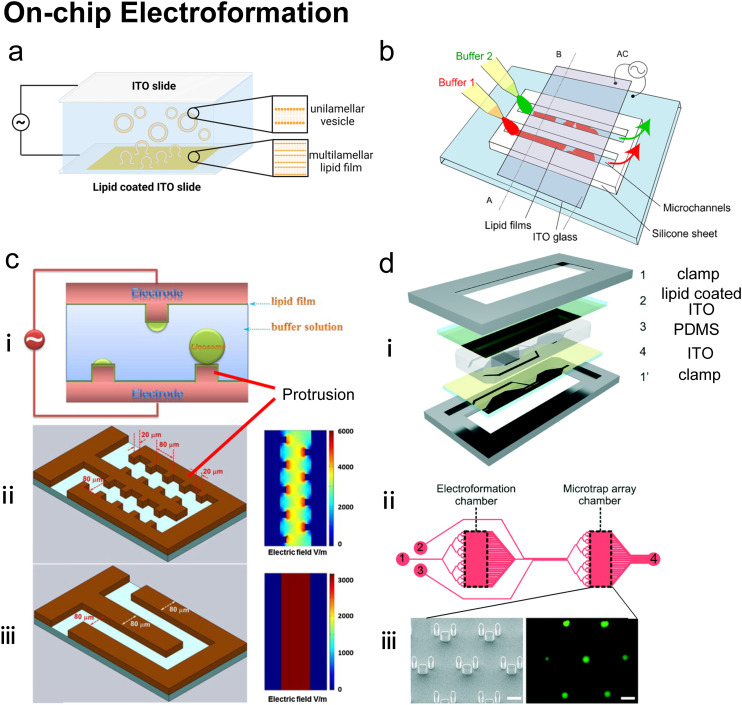
On-chip electroformation. a| Mechanism of conventional electroformation. Lipid film is coated on the surface of the electrode, usually indium tin oxide (ITO) slides. An electric field is applied across the lipid film and surrounding buffer. The lipids interact with the aqueous solution and electric field by “peeling off” the electrode surface in layers and self-assembling into vesicles. b| Schematic of electroformation in a microfluidic device developed by Kuribayashi *et al.* The glass slides were coated with ITO electrodes and clamped a silicone sheet containing microfluidic channels where the electroformation occurred. Reproduced from ref. 201 with permission from IOP Publishing, copyright [2006]. c| (i) Schematic diagram of on-chip giant vesicles electroformation process developed by Wang *et al.* (ii) Protruding microelectrode array with spatially non-uniform electric field. And (iii) planar electrode array with uniform electric field. Reproduced from ref. 202 with permission from Elsevier, copyright [2013]. d| (i) Exploded 3D diagram of microfluidic electroformation device developed by Paterson *et al.*, showing 1,1′ clamps; 2 lipid-coated ITO-coated slide; 3 PDMS sheet and 4 ITO-coated slide, arranged into a glass–PDMS–glass sandwich. (ii) Plan view of chip design (top), showing the electroformation and microtrap analysis chambers, connected by microfluidic channels (1), also depicted are the (2) wash and (3) peptide channels, as well as a collective outlet for waste (4). (iii) The microtrap array region was fabricated to capture GUVs for imaging analysis, of which the SEM image (bottom left, scale bar represents 50 μm) and fluorescent image of GUVs within it (bottom right, scale bar represents 50 μm) are presented. Reproduced from ref. 203 with permission from the Royal Society of Chemistry, copyright [2014].

Electroformation is another classic batch dispersion method for liposomal preparation. Similar to hydration, electroformation also involves forming lipid films on a solid surface and immersing the coated surface in an aqueous solution. Electroformation relies on an electric field, rather than mechanical forces, to drive the budding and self-assembly of liposomes (Fig. 14a). Electroformation has been widely used in preparing giant unilamellar vesicles.^24^

Microfluidics has been used to enhance the performance of conventional electroformation.^201–204^ Kuribayashi *et al.*^201^ (Fig. 14b) fabricated a sandwich-like microfluidic electroformation device, in which PDMS microchannels were set between two glass slides coated with indium tin oxide (ITO) electrodes. The giant vesicles formed in these microchannels were 90% unilamellar and presented good encapsulation of nanometre or micrometre-sized (200 nm or 1 μm in diameter) polystyrene beads. Le Berre *et al.*^204^ substituted one of the two ITO glass electrodes with a silicon electrode as the substrate for lipid film. Different from fabricating microchannels on PDMS as Kuribayashi *et al.*^201^ did, Le Berre *et al.*^204^ fabricated microstructure patterns on the surface of silicone electrode directly by growing the SiO_2_ layer. Both the chemical properties and topology of the Si surface exhibited an effect on vesicle size. The dimensions of the microstructure could control the size of the resultant vesicles. Similarly, Wang *et al.*^202^ (Fig. 14c) etched protruding microelectrode arrays on a heavily doped silicon wafer to generate a non-uniform electric field in the microchannel. Comparing giant vesicles formed under non-uniform and uniform electric fields, they found that the non-uniform electric field with higher electric field strength, which was caused by the protruding microelectrode arrays, could accelerate the swelling of vesicles near these microelectrodes. This non-uniform electric field was also considered to have the potential to fuse the electroformed giant vesicles. Paterson *et al.*^203^ conducted electroformation, purification and analysis of GUVs on one microfluidic platform (Fig. 14d). The GUVs produced by on-chip electroformation were trapped by micropillars so that the unencapsulated dyes were washed away. After inducing the pore-forming antimicrobial peptide melittin, leakage of fluorescent dyes could be characterized.

Compared with the emulsion-based method, electroformation is a solvent-free GUV procedure, where the problem of residual oil does not exist. Electroformation was also applied to forming GUVs containing high cholesterol percentages comparable to mammalian cells,^205^ which might be problematic for some emulsion-based methods.^204^ However, it is challenging for electroformation to form asymmetric GUVs. The internal and external buffer environments cannot be defined individually during the generation of GUVs. There are also concerns and progress about electroformation mentioned in previous reviews,^18,188^ such as the low salt concentration of buffers and the hard incorporation of membrane proteins. Generally, the involvement of microfluidics mainly enhanced electroformation in size control^201–204^ and vesicle manipulation.^203^ However, due to the batch generation of lipid films, the other advantages of microfluidics, such as high throughput and continuous production, have not been reported, which could be the direction for further improvement.

## 4 Perspectives

The broader application of liposomal drug delivery in the medical and health industry, along with the growing research interest in artificial cells and vesicle-based robotic devices, is driving the need for more advanced methods for liposome and lipid vesicle preparation.^206^ In the last two decades, microfluidic technologies have greatly benefited liposome formation. Whether completely on-chip or only partly microfluidic, the involvement of microfluidics facilitates the high throughput production of monodisperse liposomes with precise control of operational parameters. The remarkable characteristics of microfluidic liposome formation are also impressive and encouraging in producing other assemblies such as fatty acid vesicles, polymersomes, colloidosomes, and coacervates.^157,207^

There have been various branches of microfluidic technologies that produce distinct subtypes of liposomes. Different applications emphasize different liposome properties and determine the applicability of preparation methods (Table 5). Various microfluidic methods can nearly cover the production of all types of vesicles, ranging from SUVs with outstanding encapsulation efficiency for drug delivery to cell-sized vesicles with multiple compartments for mimicking the architecture of eukaryotic cells.

**Table 5:** Summary of microfluidic technologies for vesicle preparation

Preparation methods and references	Advantages	Disadvantages
Microfluidic hydrodynamic focusing (MHF):^15,68–75,77–84,86–90^	High-throughput and continuous production of nano-sized vesicles	Residual organic solvent
	Monodisperse vesicle size and lamellarity	Limited range of size and lamellarity
	High encapsulation efficiency	Low production concentration
Micromixer	High-throughput and continuous production of nano-sized vesicles	Residual organic solvent
Staggered herringbone mixer (SHM):^45,103,107–116^	High encapsulation efficiency	Less monodisperse size compared with MHF
Twisted channel (iLiNP):^119,120^	Highly commercialized	
Dean flow:^101,122,124–129^		
On-chip hydration ^134–136^	Oil-free	Batch production
	Homogeneous size controlled by microstructures	Polydisperse lamellarity
		Low encapsulation efficiency
Microfluidic refined emulsion phase transfer ^145–147^	High-throughput and continuous generation of droplets	Low-throughput batch formation of vesicles
	Monodisperse size and lamellarity	Residual oil solvent
	High encapsulation efficiency	Bursting of droplets
	Generation of membrane asymmetry	
Ice droplet hydration ^149,150^	Clean removal of organic phase	Low encapsulation efficiency
	Monodisperse vesicle size	Uncontrollable lamellarity
	Avoiding extensive droplet coalescence	
Microfluidic single emulsion transfer ^151–155^	Continuous production of cell-sized vesicles	Low-throughput vesicle formation
	High-throughput generation of droplets	Residual oil solvent
	Monodisperse size and lamellarity	Bursting of droplets
	High encapsulation efficiency	
	Adaptability for asymmetric vesicles	
Microfluidic double emulsion-based vesicle generation ^138,163,166–170,173,174,177–180^	High-throughput and continuous production of cell-sized vesicles	Residual oil solvent
	Monodisperse size and lamellarity	Sophisticated equipment
	High encapsulation efficiency	
	Adaptability for asymmetric vesicles, multi-compartmental vesicles	
Continuous droplet interface cross encapsulation (cDICE) ^181–188^	Easy fabrication and affordable device cost	Limited oil selection
	Uniform size high encapsulation efficiency	Poor partition of specific lipids
	Adaptability for asymmetric vesicles	
Pulsed jetting ^46,190–200^	High-throughput production of cell-sized vesicles	Residual oil solvent
	High encapsulation efficiency	Existence of small satellite vesicles
	Adaptability for asymmetric vesicles, multi-compartmental vesicles and nano-sized vesicles	
On-chip electroformation ^201–204^	Oil-free	Batch production
	Highly unilamellar population	Hard asymmetry construction
	Good encapsulation efficiency	Hard protein incorporation

Drug delivery requires vesicles with uniform nano-scale diameters. Continuous production with high throughput and precise size control makes microfluidics a promising direction for industrial scale-up. Among the emerging microfluidic liposomal preparation methods, MHF and micromixers have demonstrated great potential in producing lipid nanocarriers for drugs and genetic materials. Particularly, micromixers, represented by TrM and SHM, have been extensively commercialized due to their superior production rates.^111–113,116,124,125,127,129^ For further scale-up manufacturing of lipid nanocarriers, parallelized microfluidic devices are a promising solution.^45,114^ Constructing an advanced soft matter system requires cell-like or organelle-like vesicles with user-defined membrane properties such as membrane compartments and asymmetry. Emulsion-based microfluidics and pulsed jetting are suitable for constructing microscale vesicles ranging from simple GUVs to vesicles with higher-order compartments^167,168,192^ and asymmetric leaflets.^146,152,154,166,179,184,191,195^

Just as each method has its advantages, it also has its limitations, which can be mitigated by combining different techniques. For example, switching between the double emulsion and the MHF method (or combining them) on the same microfluidic device could be attempted since the two methods share similar chip geometries. In this aspect, one-step microfluidic platforms that can switch between multiple liposomal products or support simultaneous coformulations are still challenging.

To build one-step microfluidic liposomal platforms, integrating liposome formation, manipulation and analysis on a single chip is an attractive direction for the future development of microfluidic technologies.^152,208^ Rapid progress has been made in on-chip manipulation and analysis of liposomes. For instance, implementing surface tethering,^209^ optical trapping,^208,210,211^ and electric field confining^212^ has enabled the successful immobilization of vesicles on microfluidic devices and more elaborate vesicle manipulations. Filtration by microstructure,^213^ deterministic lateral displacement (DLD),^214^ pinched flow fractionation,^215^ and inertia focusing^208^ have been applied for the on-chip size-based selection of vesicles. These integrated microfluidic platforms have the potential to translate to scale-down for point-of-care applications.

To produce vesicles continuously, the continuous co-existence of two immiscible phases is indispensable. This explains why the issue of residual organic solvents trapped in the membrane, a longstanding problem in bulk liposome preparation, persists in many microfluidic-based methods. Looking ahead, further optimization of microfluidic processes is necessary to achieve effective on-chip removal of organic solvents. Supercritical fluids (SCFs) were reported to be a suitable alternative solvent for lipids for vesicle formation, which could be removed relatively easily and ensure high encapsulation efficiency.^216,217^ This could lead to combining SCFs and microfluidics if new microfluidic devices can tolerate high working pressures.

Compared with conventional bulk methods, microfluidic-based methods are usually more complex and time-consuming in designing and fabricating devices and setting up experiments. Integrating aspects of additive manufacturing, *e.g.*, 3D printing,^97,132^ as well as automation^218,219^ and machine learning^220,221^ could further revolutionise the use of microfluidic vesicle production in research, facilitating rapid device testing and optimisation.^222^ This will accelerate the uptake of microfluidic production methods in clinical and industrial applications, leading to improved delivery systems, diagnostics and microreactors.

Finally, although microfluidics presents many advantages over conventional bulk methods in forming liposomes, it does not mean microfluidic methods will completely replace conventional bulk methods. Some microfluidic methods have superseded conventional bulk methods concerning the control of parameters including size and polydispersity control. However, conventional methods for liposomal preparation, such as hydration, extrusion, emulsion phase transfer and electroformation, will remain popular in research and industry due to their simplicity of implementation. Generally, microfluidics and bulk lipid vesicle production represent two different approaches but are complementary to each other. The progress in one area can usually inspire the other area.

## Data availability

No primary research results, software or code have been included and no new data were generated or analysed as part of this review.

## Author contributions

Y. C. and C. D. H. wrote the original draft. S. M. M. reviewed the manuscript and suggested the nano-carrier part. J. W. H., Y. E. and O. C. reviewed and edited the manuscript.

## Conflicts of interest

There are no conflicts to declare.

## References

[cit1] Ryman B. E., Tyrrell D. A. (1979). Front. Biol..

[cit2] Walde P. , Preparation of vesicles (liposomes), in Encyclopedia of Nanoscience and Nanotechnology, ed. H. S. Nalwa, American Scientific Publishers, 2004, vol. 9, pp. 43–79

[cit3] Martin S. and Berg M., Biology, 10th edn, 2015, p. 107

[cit4] Kumar V. (1991). Complementary molecular shapes and additivity of the packing parameter of lipids. Proc. Natl. Acad. Sci. U. S. A..

[cit5] Bangham A. D., Horne R. W. (1964). J. Mol. Biol..

[cit6] Terama E. (2008). *et al.*, Influence of Ethanol on Lipid Membranes: From Lateral Pressure Profiles to Dynamics and Partitioning. J. Phys. Chem. B.

[cit7] McLean L. R., Phillips M. C. (1981). Mechanism of Cholesterol and Phosphatidylcholine Exchange or Transfer between Unilamellar Vesicles. Biochemistry.

[cit8] Roldán-Vargas S. (2009). *et al.*, Surface fractals in liposome aggregation. Phys. Rev. A: At., Mol., Opt. Phys..

[cit9] Verchère A. (2017). *et al.*, Reconstitution of membrane proteins in liposomes. Methods Mol. Biol..

[cit10] Rigaud J. (1988). *et al.*, Mechanisms of Membrane Protein Insertion into Liposomes during Reconstitution Procedures Involving the Use of Detergents. Biochemistry.

[cit11] Samad A., Sultana Y., Aqil M. (2007). Liposomal Drug Delivery Systems: An Update Review. Curr. Drug Delivery.

[cit12] Allen T. M., Cullis P. R. (2013). Liposomal drug delivery systems: From concept to clinical applications. Adv. Drug Delivery Rev..

[cit13] Hammer D. A., Kamat N. P. (2012). Towards an artificial cell. FEBS Lett..

[cit14] Xu C. (2016). *et al.*, Artificial cells: from basic science to applications. Mater. Today.

[cit15] Carugo D. (2016). *et al.*, Liposome production by microfluidics: potential and limiting factors. Sci. Rep..

[cit16] Tenchov R., Bird R., Curtze A. E., Zhou Q. (2021). Lipid Nanoparticles From Liposomes to mRNA Vaccine Delivery, a Landscape of Research Diversity and Advancement. ACS Nano.

[cit17] Spoelstra W. (2018). *et al.*, Tailoring the appearance: what will synthetic cells look like?. Curr. Opin. Biotechnol..

[cit18] Swaay D. V., Demello A. (2013). Microfluidic methods for forming liposomes. Lab Chip.

[cit19] Autebert J. (2012). *et al.*, Microfluidic: An innovative tool for efficient cell sorting. Methods.

[cit20] Walde P. (2010). *et al.*, Giant vesicles: preparations and applications. ChemBioChem.

[cit21] Dua J. S. (2012). *et al.*, Liposome: methods of preparation and applications. Int. J. Pharm. Sci. Res..

[cit22] Akbarzadeh A. (2013). *et al.*, Liposome: classification, preparation, and applications. Nanoscale Res. Lett..

[cit23] Walde P., Ichikawa S. (2001). Enzymes inside lipid vesicles: preparation, reactivity and applications. Biomol. Eng..

[cit24] Angelova M. I. (1992). *et al.*, Preparation of giant vesicles by external AC electric fields. Kinetics and applications. Colloid Polym. Sci..

[cit25] de Freitas C. F. (2019). *et al.*, Rapid formation of Small Unilamellar Vesicles (SUV) through low-frequency sonication: an innovative approach. Colloids Surf., B.

[cit26] Olson F., Hunt C., Szoka F., Vail W. (1979). Preparation of liposomes of defined size distribution by extrusion through polycarbonate membranes. Biochim. Biophys. Acta, Biomembr..

[cit27] Campbell M. J. (1995). Lipofection reagents prepared by a simple ethanol injection technique. BioTechniques.

[cit28] Szoka F., Papahadjopoulos D. (1978). Procedure for preparation of liposomes with large internal aqueous space and high capture by reverse-phase evaporation. Proc. Natl. Acad. Sci. U. S. A..

[cit29] Deamer D., Bangham A. D. (1976). Large volume liposomes by an ether vaporization method. Biochim. Biophys. Acta.

[cit30] Pidgeon C., McNeely S., Schmidt T., Johnson J. E. (1987). Multilayered vesicles prepared by reverse-phase evaporation: liposome structure and optimum solute entrapment. Biochemistry.

[cit31] Hamada T. (2008). *et al.*, Construction of asymmetric cell-sized lipid vesicles from lipid-coated water-in-oil microdroplets. J. Phys. Chem. B.

[cit32] Pautot S., Frisken B., Weitz D. (2003). Production of unilamellar vesicles using an inverted emulsion. Langmuir.

[cit33] Moga A. (2019). *et al.*, Optimization of the Inverted Emulsion Method for High-Yield Production of Biomimetic Giant Unilamellar Vesicles. ChemBioChem.

[cit34] Matsushita-Ishiodori Y. (2019). *et al.*, Using Imaging Flow Cytometry to Quantify and Optimize Giant Vesicle Production by Water-in-oil Emulsion Transfer Methods. Langmuir.

[cit35] Alpes H. (1986). *et al.*, Formation of large unilamellar vesicles using alkyl maltoside detergents. Biochim. Biophys. Acta.

[cit36] Fischer T. H., Lasic D. D. (1984). A detergent depletion technique for the preparation of small vesicles. Mol. Cryst. Liq. Cryst..

[cit37] Hindley J. W. (2019). *et al.*, Building a synthetic mechanosensitive signalling pathway in compartmentalized artificial cells. Proc. Natl. Acad. Sci. U. S. A..

[cit38] Trantidou T. (2018). *et al.*, Droplet microfluidics for the construction of compartmentalised model membranes. Lab Chip.

[cit39] Whitesides G. M. (2006). The Origins and the Future of Microfluidics. Nature.

[cit40] Ren K. (2013). *et al.*, Materials for microfluidic chip fabrication. Acc. Chem. Res..

[cit41] McDonald J. (2002). *et al.*, Poly(dimethylsiloxane) as a material for fabricating microfluidic devices. Acc. Chem. Res..

[cit42] Jansen H. V. (2009). *et al.*, Black silicon method: X. A review on high speed and selective plasma etching of silicon with profile control: an in-depth comparison between Bosch and cryostat DRIE processes as a roadmap to next generation equipment. J. Micromech. Microeng..

[cit43] Marre S., Jensen K. F. (2010). Synthesis of Micro and Nanostructures in Microfluidic Systems. Chem. Soc. Rev..

[cit44] Sato Y. (2019). *et al.*, Creation of artificial cell-like structures promoted by microfluidics technologies. Micromachines.

[cit45] Belliveau N. M. (2012). *et al.*, Microfluidic synthesis of highly potent limit-size lipid nanoparticles for in vivo delivery of siRNA. Mol. Ther.--Nucleic Acids.

[cit46] Funakoshi K. (2007). *et al.*, Formation of giant lipid vesicle-like compartments from a planar lipid membrane by a pulsed jet flow. J. Am. Chem. Soc..

[cit47] Goins B. A., Phillips W. T. (2001). The use of scintigraphic imaging as a tool in the development of liposome formulations. Prog. Lipid Res..

[cit48] Ferhan A. R. (2022). *et al.*, Lipid Nanoparticle Technologies for Nucleic Acid Delivery: A Nanoarchitectonics Perspective. Adv. Funct. Mater..

[cit49] Evers M. J. W. (2018). *et al.*, State-of-the-Art Design and Rapid-Mixing Production Techniques of Lipid Nanoparticles for Nucleic Acid Delivery. Small Methods.

[cit50] Gregoriadis G. (2021). Liposomes and mRNA: Two technologies together create a COVID-19 vaccine. Med. Drug Discovery.

[cit51] Strachan J. (2020). *et al.*, Toxicity and cellular uptake of lipid nanoparticles of different structure and composition. J. Colloid Interface Sci..

[cit52] Nagayasu A., Uchiyama K., Kiwada H. (1999). The size of liposomes: a factor which affects their targeting efficiency to tumours and therapeutic activity of liposomal antitumor drugs. Adv. Drug Delivery Rev..

[cit53] Longmire M., Choyke P. L., Kobayashi H. (2008). Nanomedicine.

[cit54] Betageri G. V., Parsons D. L. (1992). Drug encapsulation and release from multilamellar and unilamellar liposomes. Int. J. Pharm..

[cit55] Du Plessis J., Ramachandran C., Weiner N., Müller D. G. (1996). The influence of lipid composition and lamellarity of liposomes on the physical stability of liposomes upon storage. Int. J. Pharm..

[cit56] Maeda H., Sawa T., Konno T. (2001). Mechanism of tumour-targeted delivery of macromolecular drugs, including the EPR effect in solid tumor and clinical overview of the prototype polymeric drug SMANCS. J. Controlled Release.

[cit57] Maeki M. (2018). *et al.*, Advances in microfluidics for lipid nanoparticles and extracellular vesicles and applications in drug delivery systems. Adv. Drug Delivery Rev..

[cit58] Salim M., Minamikawa H., Sugimura A., Hashim R. (2014). Amphiphilic designer nano-carriers for controlled release: from drug delivery to diagnostics. MedChemComm.

[cit59] Mühlen A. Zur, Schwarz C., Mehnert W. (1998). Solid lipid nanoparticles (SLN) for controlled drug delivery – Drug release and release mechanism. Eur. J. Pharm. Biopharm..

[cit60] Zabara A., Mezzenga R. (2014). Controlling molecular transport and sustained drug release in lipid-based liquid crystalline mesophases. J. Controlled Release.

[cit61] Lee Y., Thompson D. (2017). Stimuli-responsive liposomes for drug delivery. Wiley Interdiscip. Rev. Nanomed. Nanobiotechnol..

[cit62] Düzgüneş N., Nir S. (1999). Mechanisms and kinetics of liposome-cell interactions. Adv. Drug Delivery Rev..

[cit63] Gandek T. (2023). *et al.*, A Comparison of Cellular Uptake Mechanisms, Delivery Efficacy, and Intracellular Fate between Liposomes and Extracellular Vesicles. Adv. Healthcare Mater..

[cit64] Sur S. (2014). *et al.*, Remote loading of preencapsulated drugs into stealth liposomes. Proc. Natl. Acad. Sci. U. S. A..

[cit65] Gubernator J. (2011). Active methods of drug loading into liposomes: Recent strategies for stable drug entrapment and increased in vivo activity. Expert Opin. Drug Delivery.

[cit66] Carvalho B. G. (2022). *et al.*, Advanced Microfluidic Technologies for Lipid Nano-Microsystems from Synthesis to Biological Application. Pharmaceutics.

[cit67] Sercombe L. (2015). *et al.*, Advances and challenges of liposome assisted drug delivery. Front. Pharmacol..

[cit68] Jahn A. (2004). *et al.*, Controlled Vesicle Self-Assembly in Microfluidic Channels with Hydrodynamic Focusing. J. Am. Chem. Soc..

[cit69] Jahn A. (2007). *et al.*, Microfluidic Directed Formation of Liposomes of Controlled Size. Langmuir.

[cit70] Jahn A. (2010). *et al.*, Microfluidic Mixing and the Formation of Nanoscale Lipid Vesicles. ACS Nano.

[cit71] Hood R., DeVoe D. (2015). *et al.*, High-Throughput Continuous Flow Production of Nanoscale Liposomes by Microfluidic Vertical Flow Focusing. Small.

[cit72] Hood R., DeVoe D. (2014). *et al.*, A facile route to the synthesis of monodisperse nanoscale liposomes using 3D microfluidic hydrodynamic focusing in a concentric capillary array. Lab Chip.

[cit73] Han J. Y., DeVoe D. (2022). *et al.*, Microfluidic vortex focusing for high throughput synthesis of size-tunable liposomes. Nat. Commun..

[cit74] Balbino T. A. (2017). *et al.*, Integrated microfluidic devices for the synthesis of nanoscale liposomes and lipoplexes. Colloids Surf., B.

[cit75] Krzysztoń R. (2017). *et al.*, Microfluidic self-assembly of folate-targeted monomolecular siRNA-lipid nanoparticles. Nanoscale.

[cit76] Lasic D. D. (1988). The Mechanism of Vesicle Formation. Biochem. J..

[cit77] Jahn A. (2013). *et al.*, Freezing continuous-flow self-Assembly in a microfluidic device: Toward imaging of liposome formation. Langmuir.

[cit78] Zook J. (2010). *et al.*, Effects of temperature, acyl chain length, and flow-rate ratio on liposome formation and size in a microfluidic hydrodynamic focusing device. Soft Matter.

[cit79] Choi S. H. (2021). *et al.*, Continuous preparation of bicelles using hydrodynamic focusing method for bicelle to vesicle transition. Micro Nano Syst. Lett..

[cit80] Pilkington C. P. (2023). *et al.*, A microfluidic platform for the controlled synthesis of architecturally complex liquid crystalline nanoparticles. Sci. Rep..

[cit81] Mijajlovic M. (2013). *et al.*, Microfluidic hydrodynamic focusing based synthesis of POPC liposomes for model biological systems. Colloids Surf., B.

[cit82] Amrani S. (2018). *et al.*, Characterization of Nanoscale Loaded Liposomes Produced by 2D Hydrodynamic Flow Focusing. ACS Biomater. Sci. Eng..

[cit83] Balbino T. A. (2013). *et al.*, Continuous flow production of cationic liposomes at high lipid concentration in microfluidic devices for gene delivery applications. Chem. Eng. J..

[cit84] Koh C. G. (2010). *et al.*, Delivery of antisense oligodeoxyribonucleotide lipopolyplex nanoparticles assembled by microfluidic hydrodynamic focusing. J. Controlled Release.

[cit85] Huang X. (2017). *et al.*, Microfluidic hydrodynamic focusing synthesis of polymer-lipid nanoparticles for siRNA delivery. Onco Targets Ther.

[cit86] Vladisavljevic G. (2014). *et al.*, Production of liposomes using microengineered membrane and co-flow microfluidic device. Colloids Surf., A.

[cit87] Lin W. (2019). *et al.*, Liposome production and concurrent loading of drug simulants by microfluidic hydrodynamic focusing. Eur. Biophys. J..

[cit88] Pilkington C. (2024). *et al.*, Engineering a nanoscale liposome-in-liposome for in situ biochemical synthesis and multi-stage release. Nat. Chem..

[cit89] Kim Y. (2012). *et al.*, Single Step Reconstitution of Multifunctional High Density Lipoprotein Derived Nanomaterials Using Microfluidics. Nano Lett..

[cit90] Modarres P., Tabrizian M. (2020). Phase-controlled field-effect micromixing using AC electroosmosis. Microsyst. Nanoeng..

[cit91] Thiele J. (2010). *et al.*, Preparation of monodisperse block copolymer vesicles via flow focusing in microfluidics. Langmuir.

[cit92] Valencia P. M. (2010). *et al.*, Single-step assembly of homogeneous lipid-polymeric and lipid-quantum dot nanoparticles enabled by microfluidic rapid mixing. ACS Nano.

[cit93] Zhu C. (2018). *et al.*, Self-assembly of fluorinated gradient copolymer in three-dimensional co-flow focusing microfluidic. J. Colloid Interface Sci..

[cit94] Zizzari A. (2022). *et al.*, Environmentally Friendly Method of Assembly of Cardanol and Cholesterol into Nanostructures Using a Continuous Flow Microfluidic Device. ACS Sustainable Chem. Eng..

[cit95] Puigmartí-Luis J. (2010). *et al.*, A microfluidic approach for the formation of conductive nanowires and hollow hybrid structures. Adv. Mater..

[cit96] Lu M. (2016). *et al.*, Microfluidic hydrodynamic focusing for synthesis of nanomaterials. Nano Today.

[cit97] Hampson S. M. (2018). *et al.*, 3D printed microfluidic device with integrated optical sensing for particle analysis. Sens. Actuators, B.

[cit98] Bayram A. (2018). *et al.*, Integration of glass micropipettes with a 3D printed aligner for microfluidic flow cytometer. Sens. Actuators, A.

[cit99] Gursoy A. (2020). *et al.*, Facile fabrication of microfluidic chips for 3D hydrodynamic focusing and wet spinning of polymeric fibers. Polymers.

[cit100] Cinquerrui S. (2018). *et al.*, Nanoencapsulation of bacteriophages in liposomes prepared using microfluidic hydrodynamic flow focusing. Front. Microbiol..

[cit101] López R. R. (2021). *et al.*, Parametric Study of the Factors Influencing Liposome Physicochemical Characteristics in a Periodic Disturbance Mixer. Langmuir.

[cit102] Nguyen N. T. , Micromixers fundamentals, design and fabrication, William Andrew, Amsterdam, 2nd edn, 2012, ch. 2

[cit103] Zhigaltsev I. V. (2012). *et al.*, Bottom-up design and synthesis of limit size lipid nanoparticle systems with aqueous and triglyceride cores using millisecond microfluidic mixing. Langmuir.

[cit104] Cartwright J. H. E. , Feingold M. and Piro O., An Introduction to Chaotic Advection, 1999

[cit105] Aref H. (1984). Stirring by chaotic advection. J. Fluid Mech..

[cit106] Stroock A. D. (2002). *et al.*, Chaotic mixer for microchannels. Science.

[cit107] Zhigaltsev I. V. (2016). *et al.*, Production of limit size nanoliposomal systems with potential utility as ultra-small drug delivery agents. J. Liposome Res..

[cit108] Maeki M. (2015). *et al.*, A strategy for synthesis of lipid nanoparticles using microfluidic devices with a mixer structure. RSC Adv..

[cit109] Maeki M. (2017). *et al.*, Understanding the formation mechanism of lipid nanoparticles in microfluidic devices with chaotic micromixers. PLoS One.

[cit110] Cheung C. C. L. (2019). *et al.*, Sterically stabilized liposomes production using staggered herringbone micromixer: Effect of lipid composition and PEG-lipid content. Int. J. Pharm..

[cit111] Shah V. M. (2019). *et al.*, Liposomes produced by microfluidics and extrusion: A comparison for scale-up purposes. Nanomedicine.

[cit112] Joshi S. (2016). *et al.*, Microfluidics based manufacture of liposomes simultaneously entrapping hydrophilic and lipophilic drugs. Int. J. Pharm..

[cit113] Kastner E. (2015). *et al.*, High-throughput manufacturing of size-tuned liposomes by a new microfluidics method using enhanced statistical tools for characterization. Int. J. Pharm..

[cit114] Shepherd S. J. (2021). *et al.*, Scalable mRNA and siRNA Lipid Nanoparticle Production Using a Parallelized Microfluidic Device. Nano Lett..

[cit115] Leung K. K. (2012). *et al.*, Lipid nanoparticles containing siRNA synthesized by microfluidic mixing exhibit an electron-dense nanostructured core. J. Phys. Chem..

[cit116] Elsana H. (2019). *et al.*, Evaluation of novel cationic gene based liposomes with cyclodextrin prepared by thin film hydration and microfluidic systems. Sci. Rep..

[cit117] Love K. T. (2010). *et al.*, Lipid-like materials for low-dose, in vivo gene silencing. Proc. Natl. Acad. Sci. U. S. A..

[cit118] Semple S. C. (2010). *et al.*, Rational design of cationic lipids for siRNA delivery. Nat. Biotechnol..

[cit119] Kimura N. (2018). *et al.*, Development of the iLiNP Device: Fine Tuning the Lipid Nanoparticle Size within 10 nm for Drug Delivery. ACS Omega.

[cit120] Suzuki Y. (2021). *et al.*, Lipid nanoparticles loaded with ribonucleoprotein–oligonucleotide complexes synthesized using a microfluidic device exhibit robust genome editing and hepatitis B virus inhibition. J. Controlled Release.

[cit121] Nivedita N. (2017). *et al.*, Dean Flow Dynamics in Low-Aspect Ratio Spiral Microchannels. Sci. Rep..

[cit122] Lee J. (2013). *et al.*, High-throughput nanoscale lipid vesicle synthesis in a semicircular contraction-expansion array microchannel. BioChip J..

[cit123] Shi H. (2020). *et al.*, Numerical investigation of the secondary flow effect of lateral structure of micromixing channel on laminar flow. Sens. Actuators, B.

[cit124] Webb C., Perrie Y. (2020). *et al.*, Using microfluidics for scalable manufacturing of nanomedicines from bench to GMP: A case study using protein-loaded liposomes. Int. J. Pharm..

[cit125] Ripoll M. (2022). *et al.*, Optimal self-assembly of lipid nanoparticles (LNP) in a ring micromixer. Sci. Rep..

[cit126] López R. (2020). *et al.*, Surface response-based modelling of liposome characteristics in a periodic disturbance mixer. Micromachines.

[cit127] Santhanes D. (2022). *et al.*, Microfluidic formulation of lipid/polymer hybrid nanoparticles for plasmid DNA (pDNA) delivery. Int. J. Pharm..

[cit128] Valencia P. M. (2010). *et al.*, Single-step assembly of homogeneous lipid-polymeric and lipid-quantum dot nanoparticles enabled by microfluidic rapid mixing. ACS Nano.

[cit129] Roces C. B., Perrie Y. (2020). *et al.*, Manufacturing considerations for the development of lipid nanoparticles using microfluidics. Pharmaceutics.

[cit130] https://www.precisionnanosystems.com/platform-technologies/nxgen

[cit131] Firmino P. (2021). *et al.*, 3D micromixer for nanoliposome synthesis: a promising advance in high mass productivity. Lab Chip.

[cit132] Bokare A. (2019). *et al.*, Herringbone-Patterned 3D-Printed Devices as Alternatives to Microfluidics for Reproducible Production of Lipid Polymer Hybrid Nanoparticles. ACS Omega.

[cit133] MacDonald R. C., Jones F. D., Qui R. (1994). Fragmentation into small vesicles of dioleoylphosphatidylcholine bilayers during freezing and thawing. Biochim. Biophys. Acta, Biomembr..

[cit134] Lin Y., Huang K., Chiang J., Yang C., Lai T. (2006). Manipulating self-assembled phospholipid microtubes using microfluidic technology. Sens. Actuators, B.

[cit135] Suzuki H. (2008). Size Characteristics of Liposomes Formed in a Micro-Tube. J. Chem. Eng. Jpn..

[cit136] Kitazoe K. (2011). *et al.*, A touch-and-go lipid wrapping technique in microfluidic channels for rapid fabrication of multifunctional envelope-type gene delivery nanodevices. Lab Chip.

[cit137] Hindley J. W. (2020). *et al.*, Membrane functionalization in artificial cell engineering. SN Appl. Sci..

[cit138] Deshpande S. (2019). *et al.*, Spatiotemporal control of coacervate formation within liposomes. Nat. Commun..

[cit139] Aufinger L., Simmel L. F. C. (2019). Establishing Communication Between Artificial Cells. Chem. – Eur. J..

[cit140] Zhao J. (2022). *et al.*, Mimicking Cellular Metabolism in Artificial Cells: Universal Molecule Transport across the Membrane through Vesicle Fusion. Anal. Chem..

[cit141] Crane J. M. (2005). *et al.*, Measuring Lipid Asymmetry in Planar Supported Bilayers by Fluorescence Interference Contrast Microscopy. Langmuir.

[cit142] Elani Y., Law R. V., Ces O. (2014). Vesicle-based artificial cells as chemical microreactors with spatially segregated reaction pathways. Nat. Commun..

[cit143] Yu W. (2001). *et al.*, Synthesis of functional protein in liposome. J. Biosci. Bioeng..

[cit144] Berhanu S., Ueda T., Kuruma Y. (2019). Artificial photosynthetic cell producing energy for protein synthesis. Nat. Commun..

[cit145] Tan Y., Hettiarachchi K., Siu M., Pan Y., Lee A. (2006). Controlled microfluidic encapsulation of cells, proteins, and microbeads in lipid vesicles. J. Am. Chem. Soc..

[cit146] Romanov V. (2019). *et al.*, A Tunable Microfluidic Device Enables Cargo Encapsulation by Cell- or Organelle-Sized Lipid Vesicles Comprising Asymmetric Lipid Bilayers. Adv. Biosyst..

[cit147] Nishimura K. (2012). *et al.*, Size control of giant unilamellar vesicles prepared from inverted emulsion droplets. J. Colloid Interface Sci..

[cit148] Huang Y. (2017). *et al.*, Emulsion templated vesicles with symmetric or asymmetric membranes. Adv. Colloid Interface Sci..

[cit149] Sugiura S., Kuroiwa T. (2008). *et al.*, Novel Method for Obtaining Homogeneous Giant Vesicles from a Monodisperse Water-in-Oil Emulsion Prepared with a Microfluidic Device. Langmuir.

[cit150] Kuroiwa T., Kiuchi H. (2009). *et al.*, Controlled preparation of giant vesicles from uniform water droplets obtained by microchannel emulsification with bilayer-forming lipids as emulsifier. Microfluid. Nanofluid..

[cit151] Matosevic S., Paegel B. M. (2011). Stepwise Synthesis of Giant Unilamellar Vesicles on a Microfluidic Assembly Line. J. Am. Chem. Soc..

[cit152] Matosevic S., Paegel B. M. (2013). Layer-by-layer cell membrane assembly. Nat. Chem..

[cit153] Karamdad K. (2015). *et al.*, Preparation and mechanical characterisation of giant unilamellar vesicles by a microfluidic method. Lab Chip.

[cit154] Karamdad K. (2016). *et al.*, Studying the effects of asymmetry on the bending rigidity of lipid membranes formed by microfluidics. Chem. Commun..

[cit155] Weiss M. (2018). *et al.*, Sequential bottom-up assembly of mechanically stabilized synthetic cells by microfluidic. Nat. Mater..

[cit156] Ip T. (2021). *et al.*, Manufacture of Multilayered Artificial Cell Membranes through Sequential Bilayer Deposition on Emulsion Templates. ChemBioChem.

[cit157] Robinson T. (2019). Microfluidic Handling and Analysis of Giant Vesicles for Use as Artificial Cells: A Review. Adv. Biosyst..

[cit158] Bao P. (2019). *et al.*, Lipid coated liquid crystal droplets for the on-chip detection of antimicrobial peptide. Lab Chip.

[cit159] Hwang W. L. (2008). *et al.*, Asymmetric droplet interface bilayers. J. Am. Chem. Soc..

[cit160] Seifriz W. (1925). Studies in emulsions. J. Phys. Chem..

[cit161] Okushima S. (2004). *et al.*, Controlled Production of Monodisperse Double Emulsions by Two-Step Droplet Breakup in Microfluidic Devices. Langmuir.

[cit162] Abate A. R., Weitz D. A. (2009). High-order multiple emulsions formed in poly (dimethylsiloxane) microfluidics. Small.

[cit163] Shum H. C. (2008). *et al.*, Double emulsion templated monodisperse phospholipid vesicles. Langmuir.

[cit164] Shum H. C. (2011). *et al.*, Dewetting-induced membrane formation by adhesion of amphiphile-laden interfaces. J. Am. Chem. Soc..

[cit165] Shum H. C. (2011). *et al.*, Multicompartment Polymersomes from Double Emulsions. Am. Ethnol..

[cit166] Arriage L. R. (2019). *et al.*, Single-step assembly of asymmetric vesicles Laura. Lab Chip.

[cit167] Deng N. N. (2016). *et al.*, Monodisperse Uni- and Multicompartment Liposomes. J. Am. Chem. Soc..

[cit168] Deng N. N. (2017). *et al.*, Microfluidic Assembly of Monodisperse Vesosomes as Artificial Cell Models. J. Am. Chem. Soc..

[cit169] Deshpande S., Dekker C. (2018). On-chip microfluidic production of cell-sized liposomes. Nat. Protoc..

[cit170] Deng N., Huck W. T. S. (2017). MicrofluidicFormation of MonodisperseCoacervate Organelles in Liposomes. Angew. Chem..

[cit171] Deng N. N. (2020). Complex coacervates as artificial membraneless organelles and protocells. Biomicrofluidics.

[cit172] Hondele M. (2020). *et al.*, Membraneless organelles: phasing out of equilibrium. Emerging Top. Life Sci..

[cit173] Deshpande S. (2016). *et al.*, Octanol-assisted liposome assembly on chip. Nat. Commun..

[cit174] Schaich M. (2020). *et al.*, Characterization of lipid composition and diffusivity in OLA generated vesicles. Biochim. Biophys. Acta, Biomembr..

[cit175] Deshpande S. (2018). Mechanical Division of Cell-Sized Liposomes. ACS Nano.

[cit176] Tivony R. (2021). *et al.*, A Microfluidic Platform for Sequential Assembly and Separation of Synthetic Cell Models. ACS Synth. Biol..

[cit177] Last M. G. F. (2020). *et al.*, pH-Controlled Coacervate-Membrane Interactions within Liposomes. ACS Nano.

[cit178] Teh S. Y. (2011). *et al.*, Stable, biocompatible lipid vesicle generation by solvent extraction-based droplet microfluidics. Biomicrofluidics.

[cit179] Lu L. (2015). *et al.*, Continuous microfluidic fabrication of synthetic asymmetric vesicles. Lab Chip.

[cit180] Kong L. (2020). *et al.*, Lipid-Stabilized Double Emulsions Generated in Planar Microfluidic Devices. Langmuir.

[cit181] Abkarian M. (2011). *et al.*, Continuous droplet interface crossing encapsulation (cDICE) for high throughput monodisperse vesicle design. Soft Matter.

[cit182] Keber F. (2014). *et al.*, Topology and dynamics of active nematic vesicles. Science.

[cit183] Dürre K., Bausch A. R. (2019). Formation of phase separated vesicles by double layer cDICE. Soft Matter.

[cit184] Morita M. (2015). *et al.*, Droplet-Shooting and Size-Filtration (DSSF) Method for Synthesis of Cell-Sized Liposomes with Controlled Lipid Compositions. ChemBioChem.

[cit185] Van De Cauter L. (2021). *et al.*, Optimized cDICE for Efficient Reconstitution of Biological Systems in Giant Unilamellar Vesicles. ACS Synth. Biol..

[cit186] Blosser M. (2016). *et al.*, cDICE method produces giant lipid vesicles under physiological conditions of charged lipids and ionic solutions. Soft Matter.

[cit187] Deek J. (2018). *et al.*, Reconstitution of composite actin and keratin networks in vesicles. Soft Matter.

[cit188] Baldauf L. (2023). *et al.*, Branched actin cortices reconstituted in vesicles sense membrane curvature. Biophys. J..

[cit189] Elani Y., Solvas X. C. I., Edel J. B., Law R. V., Ces O. (2016). Microfluidic generation of encapsulated droplet interface bilayer networks (multisomes) and their use as cell-like reactors. Chem. Commun..

[cit190] Stachowiak J. (2008). *et al.*, Unilamellar vesicle formation and encapsulation by microfluidic jetting. Proc. Natl. Acad. Sci. U. S. A..

[cit191] Kamiya K. (2016). *et al.*, The investigation of asymmetric membranes. Nat. Chem..

[cit192] Kamiya K., Osaki T., Takeuchi S. (2019). Formation of vesicles-in-a-vesicle with asymmetric lipid components using a pulsed-jet flow method. RSC Adv..

[cit193] Belardi B. (2019). *et al.*, Claudin-4 Reconstituted in Unilamellar Vesicles Is Sufficient to Form Tight Interfaces That Partition Membrane Proteins. J. Cell Sci..

[cit194] Gotanda M., Kamiya K. (2018). *et al.*, Sequential generation of asymmetric lipid vesicles using a pulsed-jetting method in rotational wells. Sens. Actuators, B.

[cit195] Kamiya K. , *et al.*, Reconstitution and Function of Membrane proteins Into Asymmetric Giant Liposomes by Using a Pulsed Jet Flow Kanagawa Academy of Science and Technology, Japan MEMS 2014, San Francisco, CA, USA, January 26–30, 2014.288-289

[cit196] Richmond D. L. (2011). *et al.*, Forming Giant Vesicles with Controlled Membrane Composition, Asymmetry, and Contents. Proc. Natl. Acad. Sci. U. S. A..

[cit197] Gotanda M., Kamiya K. (2019). *et al.*, Automatic generation system of cell-sized liposomes. Sens. Actuators, B.

[cit198] Armstrong M. (2020). *et al.*, Forming and loading giant unilamellar vesicles with acoustic jetting. Biomicrofluidics.

[cit199] Kamiya K. (2021). Formation of nano-sized lipid vesicles with asymmetric lipid components using a pulsed-jet flow method. Sens. Actuators, B.

[cit200] Kamiya K. (2020). Development of Artificial Cell Models Using Microfluidic Technology and Synthetic Biology. Micromachines.

[cit201] Kuribayashi K. (2006). *et al.*, Electroformation of giant liposomes in microfluidic channels. Meas. Sci. Technol..

[cit202] Wang Z. (2013). *et al.*, Electroformation and electrofusion of giant vesicles in a microfluidic device. Colloids Surf., B.

[cit203] Paterson D. J. (2014). *et al.*, Integrating microfluidic generation, handling and analysis of biomimetic giant unilamellar vesicles. Lab Chip.

[cit204] Berre Le (2008). *et al.*, Electroformation of giant phospholipid vesicles on a silicon substrate: advantages of controllable surface properties. Langmuir.

[cit205] Méléard P. (1997). *et al.*, Bending elasticities of model membranes. Influences of temperature and cholesterol content. Biophys. J..

[cit206] Peng Z. (2024). *et al.*, Lipid vesicle-based molecular robots. Lab Chip.

[cit207] Spoelstra W. K., Deshpande S., Dekker C. (2018). Tailoring the appearance: what will synthetic cells look like?. Curr. Opin. Biotechnol..

[cit208] Yandrapalli N., Robinson T. (2019). Ultra-high capacity microfluidic trapping of giant vesicles for high-throughput membrane studies. Lab Chip.

[cit209] Hentrich C., Szostak J. W. (2014). Controlled growth of filamentous fatty acid vesicles under flow. Langmuir.

[cit210] Vivek A. (2020). *et al.*, Fusing Artificial Cell Compartments and Lipid Domains Using Optical Traps: A Tool to Modulate Membrane Composition and Phase Behaviour. Micromachines.

[cit211] Friddin M. S. (2019). *et al.*, Direct manipulation of liquid ordered lipid membrane domains using optical traps. Commun. Chem..

[cit212] Lee S. J., Rhee H., Jeon T.-J., Kim D. (2016). Preconcentration of lipid vesicles using concentration polarization in a microfluidic chip. Sens. Actuators, B.

[cit213] Robinson T. (2013). *et al.*, Microfluidic trapping of giant unilamellar vesicles to study transport through a membrane pore. Biomicrofluidics.

[cit214] Kazayama Y. (2016). *et al.*, Integrated Microfluidic System for Size-Based Selection and Trapping of Giant Vesicles. Anal. Chem..

[cit215] Storslett K. J., Muller S. J. (2017). Evaluation and comparison of two microfluidic size separation strategies for vesicle suspensions. Biomicrofluidics.

[cit216] Santo I. E. (2014). *et al.*, Liposomes preparation using a supercritical fluid assisted continuous process. Chem. Eng. J..

[cit217] Campardelli R., Trucillo P., Reverchon E. (2016). A Supercritical Fluid-Based Process for the Production of Fluorescein-Loaded Liposomes. Ind. Eng. Chem. Res..

[cit218] Melin J., Quake S. R. (2007). Microfluidic large-scale integration: The evolution of design rules for biological automation. Annu. Rev. Biophys. Biomol. Struct..

[cit219] Au A. K. (2015). *et al.*, 3D-printed microfluidic automation. Lab Chip.

[cit220] McIntyre D. (2022). *et al.*, Machine learning for microfluidic design and control. Lab Chip.

[cit221] Dabbagh S. R. (2020). *et al.*, Machine learning-enabled multiplexed microfluidic sensors. Biomicrofluidics.

[cit222] Friddin M. S. (2019). *et al.*, New Directions for Artificial Cells Using Prototyped Biosystems. Anal. Chem..

